# Spatial patterns of cattle densities across the Brazilian Amazon revealed by very high-resolution satellite imagery

**DOI:** 10.1038/s44458-026-00082-2

**Published:** 2026-06-16

**Authors:** Leonie Hodel, Jan D. Wegner, Vivien Sainte Fare Garnot, Francisco Carlos da Rocha Gomes, Judson Ferreira Valentim, Rachael D. Garrett

**Affiliations:** 1 Department of Geography, University of Cambridge, Cambridge, United Kingdom; 2 Conservation and Development Lab, Conservation Research Institute, University of Cambridge, Cambridge, United Kingdom; 3 EcoVision Lab, Department of Mathematical Modeling and Machine Learning (DM3L), Universitat Zurich, Zurich, Switzerland; 4 Embrapa Acre, Rio Branco, Brazil

**Keywords:** Sustainability, Environmental impact

## Abstract

Cattle ranching is a sustainability challenge worldwide, and in the Amazon, the planet’s largest tropical forest, it remains the main driver of deforestation. Yet, cattle numbers have typically been estimated from coarse census data or indirect proxies, limiting our ability to monitor land-use change at finer scales. Here, we introduce a novel approach that applies deep learning-based density estimation to very high-resolution satellite imagery to detect individual animals across the Brazilian Amazon. Our cattle data set covers over 12,000 km² in four states and is integrated with pasture maps to analyze property-level stocking rates. We find patterns of extensive land use, deriving conservative stocking rate estimates of 0.73 head per hectare in 2018–2019, with lower cattle stocking rates on properties with higher recent deforestation and properties further away from slaughterhouses. While the use of VHR imagery presents challenges of coverage and detection, our framework establishes a foundation for advancing livestock monitoring and supports strategies to address deforestation and promote sustainable resource management.

## Abstract (web summary)

Cattle stocking rates across the Brazilian Amazon are variable and lower on properties with recent deforestation or remote from slaughterhouses, according to a deep-learning approach that detects individual animals in very high-resolution satellite imagery.

## Introduction

Tropical deforestation accounts for a substantial share of global greenhouse gas (GHG) emissions and is associated with a wide range of additional environmental and social impacts^1,2^. The majority of this forest loss is associated with agricultural expansion^3^, with resulting commodities frequently destined for global markets and integrated into the supply chains of large corporate conglomerates^4,5^. In response, satellite-based monitoring systems have been widely adopted to map forest clearing, strengthening environmental law enforcement^6^, and enabling deforestation monitoring within global supply chains^7,8^. Recently, various Artificial Intelligence (AI)-based innovations have enhanced the monitoring of these land systems, including detailed maps of vegetation structure and agricultural land to foster efficiency and sustainable practices^9,10^. Yet, in the livestock sector, the largest driver of deforestation worldwide^11^ and a pivotal component of global land system sustainability^12^, satellite-based monitoring capabilities and associated research remain less developed. Existing global and regional livestock mapping efforts typically rely on downscaling methods^13,14^, costly and time-intensive field assessments, or agricultural censuses^15^. Consequently, the lack of scalable livestock monitoring severely constrains our ability to analyze land-use practices and their environmental impacts, particularly in remote deforestation frontiers where data gaps are greatest^16,17^.

Here, we leverage recent technological advances to address this limitation. Novel, very high-resolution (VHR, <1 m spatial resolution) satellite sensors enable the acquisition of observational data at large scales, opening the possibility to visualize and count individual cattle from space^18^. Additionally, AI, or more specifically, neural network-based methods, have proven their capability in processing and automatically analyzing such imagery data^19^. Convolutional neural network (CNN)-based object detection models have already been used on VHR satellite imagery data to detect and count different animal species^20–22^. More recently, other types of CNN architectures called density estimators could further advance the field^23,24^, offering two crucial advantages over the more commonly used object detectors: they allow object counts to be estimated even when individual objects occupy only a few pixels and when they occur in close proximity to each other^25^. Cattle on VHR images occupy only 3–6 pixels and frequently appear in crowded herds, making density estimators well-suited for this task^26^.

Our study region is the Brazilian Amazon, where more than 90% of deforested areas are converted into pasture^2,27^. Reducing the environmental impacts from forest conversion in the cattle sector has been a crucial component of Brazilian national climate and biodiversity strategies^28,29^, as well as sector-specific supply chain commitments^30^. Despite decades of research and policy, the decoupling of cattle production from deforestation remains unachieved^31^. Meanwhile, extensive practices and degrading pastures reduce the pasture carrying capacity, further inciting the demand for new land^32,33^. This is despite substantial evidence that improvements in cattle management could simultaneously increase farm incomes and food production while reducing pressure on forests^34–36^. Brazil’s ecological conditions would allow cattle production to be intensified to ~3–4 animal units (AU) per hectare^35,37^, yet current stocking rates remain far below, averaging around 0.91 AU nationally^34^ and between 0.73 and 1.26 AU across Amazonian States^38–40^. A more spatially explicit understanding of where and how cattle production operates and how it interacts with deforestation dynamics, market access, and policy interventions could enable more targeted strategies for sustainable intensification.

To derive cattle numbers across pasture landscapes, we employ a congested scene recognition (CSR)-based density estimator^25,26^ on VHR satellite imagery. We first validate image-derived cattle counts against field observations and assess model performance across four states in the Brazilian Amazon. We then generate cattle density maps covering more than 12,000 km² and quantify cattle densities across these regions. By integrating property boundaries and land cover data, we examine the relationship between stocking rates in 2018–2019, deforestation, and other land uses. Specifically, we address two questions: (*i*) how cattle densities vary across the Brazilian Amazon and which land-management characteristics typify cattle-producing farms?; and (*ii*) which land-use factors are associated with variation in stocking rates?

For our second question, we seek to shed light on how land-use dynamics, market integration, and cattle stocking rates interact in Amazonian cattle ranching^3,41^ using a fixed-effects regression model at the property level. Specifically, we test whether farms that have cleared forest for pasture exhibit higher stocking rates, potentially reflecting the temporary fertility boost of newly deforested soils^42^, or instead lower stocking rates, either because capital is diverted from intensification toward land expansion^43^, or because ranching is pursued primarily as a strategy for land speculation rather than productive use^3,44,45^. We explore the role of market access in cattle ranching, testing whether proximity to slaughterhouses is associated with higher cattle stocking rates through reduced transaction costs, facilitating market integration^46^.

## Results

### Advanced remote sensing methods for cattle density and stocking rate estimation

**Fig. 1:**
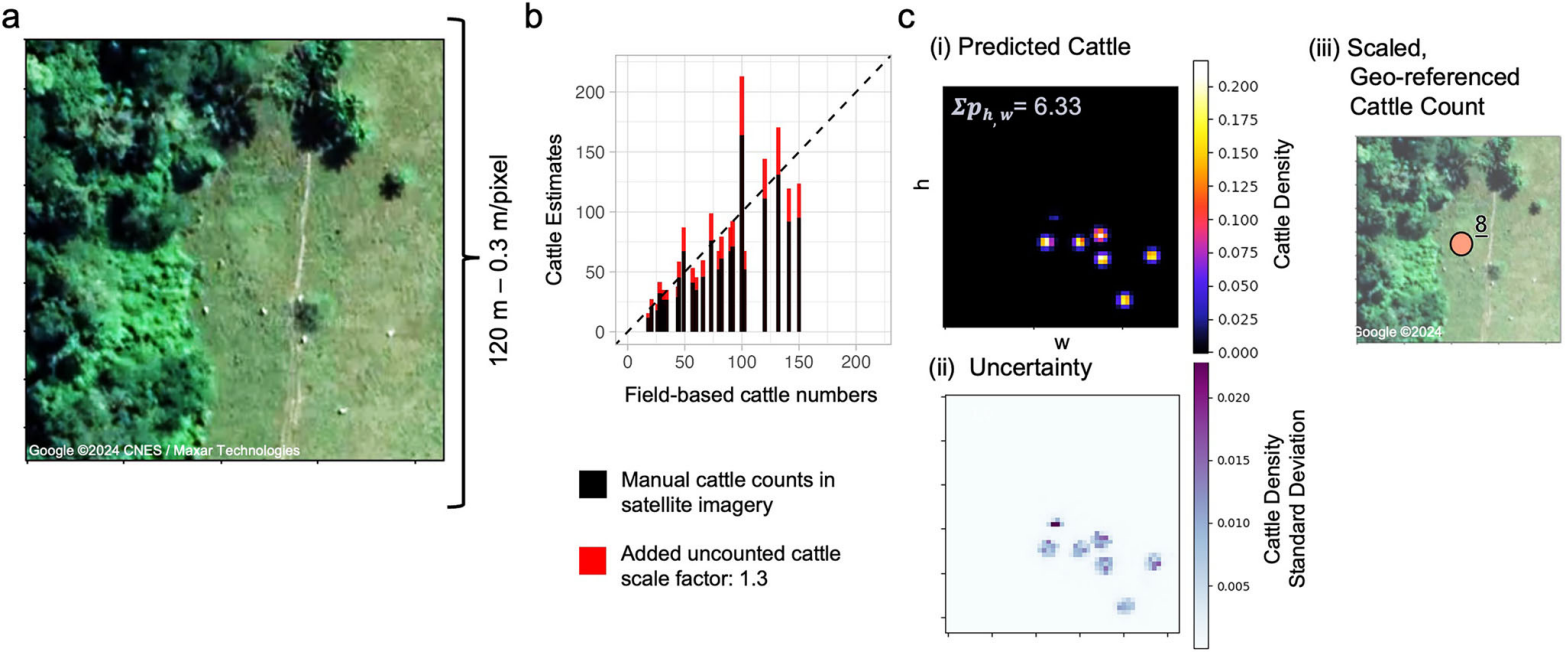
Congested scene recognition (CSR) model for cattle detection on very high-resolution (VHR) satellite imagery in the Brazilian Amazon. **a** A sample of a 120 m × 120 m and 0.3 meter resolution satellite image patch showing individual cattle. **b** Comparison of field-based cattle numbers and cattle counts in satellite images of 29 farms and 2155 animal heads. The dashed 1:1 reference line indicates perfect agreement. The CSR estimates are scaled by a factor of 1.3 to account for invisible and occluded animals in the imagery. **c** Output of the CSR model, using an ensemble approach: (i) predicted cattle heatmap for which the sum of the pixels p is 6.33 and (ii) an uncertainty measure based on the ensemble standard deviation; (iii) the sum of the predicted cattle is converted into a scaled and rounded geo-referenced cattle count. Images: Google ©2025 CNES/Airbus, Maxar Technologies.

To assess the reliability of cattle detection in VHR satellite imagery, we validated image-derived cattle counts (Fig. 1a) against field-based observations. Field data were obtained from the Agricultural and Forestry Research Institute in Acre (Portuguese: Instituto de Defesa Agropecuária e Florestal do Estado do Acre (IDAF)) and survey data^47^ across 29 cattle farms, resulting in a total of 2155 individual cattle observations in five different VHR images and production contexts in the state of Acre (Supplementary Data S1). Cattle production systems in the Amazon are predominantly extensive, characterized by open pasture areas with little tree cover (~1-2 trees per hectare^48^) and limited structures available for livestock shelter. Nonetheless, a fraction of animals is expected to be occluded in the imagery^49–51^. In our validation, we observed an overall image-to-field cattle detection rate of 77%. To account for undetected animals, we adjusted image-based cattle counts with a scaling factor of 1.3 (Fig. 1b, Supplementary Data S1).

The CSR-based density estimation model operates on image tiles of ~120 × 120 m and produces both cattle count estimates and an ensemble-based uncertainty measure (Fig. 1c). The model was trained on a data set of 6284 tiles and 18,400 labeled cattle. We estimated the model performance on an independent test set spanning a range of cattle densities, regions, and imagery sources (Supplementary Figs. 1, S2). The total Mean Absolute Error $({MAE})$ was 0.30, relative to an average density of 0.89 cattle per hectare of pasture. Error rates are comparable across regions (*MAE*_*Acre*_ = 0.28, *MAE*_*Amazonas*_ = 0.41, *MAE*_*Pará*_ = 0.44, *MAE*_*Rondônia*_ = 0.31), indicating that the model generalizes well across the study area.

**Fig. 2:**
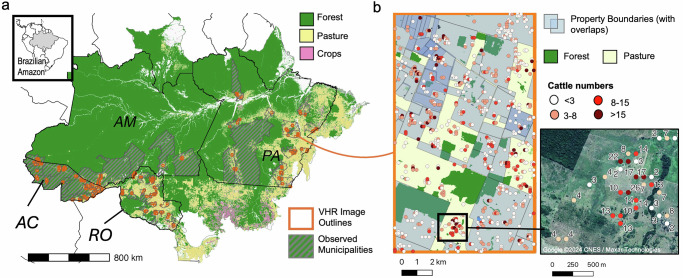
Cattle densities in four states located in the Brazilian Amazon. **a** Map of the Brazilian Amazon biome showing the outlines of 170 satellite images (in orange) across pasture landscapes (yellow) covering 12,260 km^2^. The data set covers 56 municipalities (striped) in the states of Acre (AC), Amazonas (AM), Pará (PA), and Rondônia (RO). **b** Example of a single VHR image outline: The predicted cattle densities show the presence of cattle inside and outside of property boundaries of the Environmental Rural Registry (Portuguese: Cadastro Ambiental Rural, CAR). Image: Google ©2025 CNES/Airbus, Maxar Technologies.

We then produced cattle densities with a 120 m resolution across four states in the Brazilian Amazon using 170 VHR satellite images with a total coverage of 12,260 km² (Supplementary Data S2). The VHR imagery was captured in the dry seasons of 2018–2019 (with some additional imagery from 2017, 2020, and 2021, not used in subsequent analysis). The image outlines and dates were manually annotated in Google Earth (Fig. 2a). Overlaying these maps with MapBiomas land-cover data identified 7297 km² of pasture. With a total of 367,511 cattle, and after applying the scale factor of 1.3, this results in an overall stocking rate ${SR}$ of 0.65 cattle per hectare of pasture (Supplementary Data S3). However, cattle distributions show differences according to their property registration status (Fig. 2b). Using property boundaries from the Brazilian Rural Environmental Registry (Portuguese: Cadastro Ambiental Rural, CAR)^52^, we estimate that 13% of detected cattle were located on pasture outside registered properties.

### Low property-level stocking rates, pasture degradation, and sparse uptake of integrated crop and livestock systems on cattle farms

**Fig. 3:**
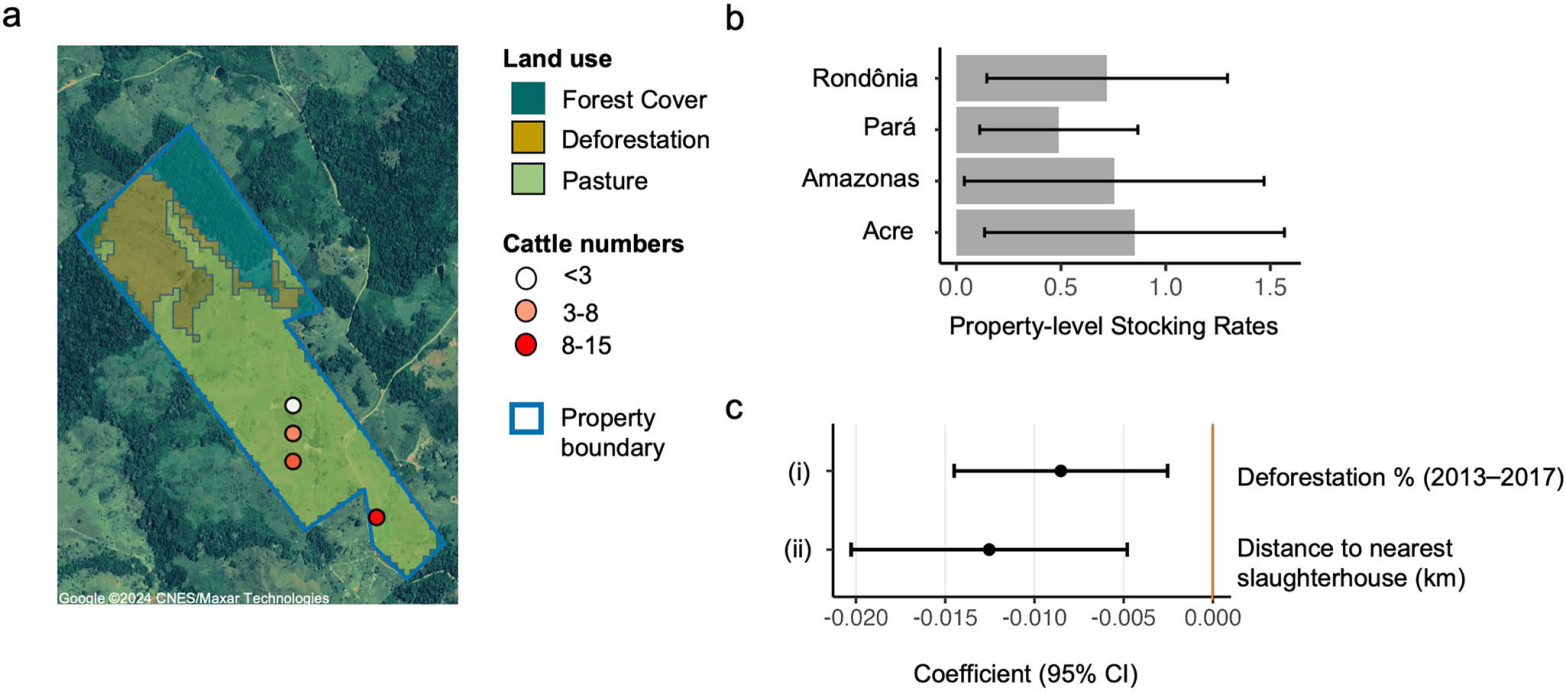
Estimation and analysis of property-level cattle stocking rates (animals per hectare pasture) of the states of Rondônia, Pará, Amazonas, and Acre in the Brazilian Amazon in 2018-2019. **a** Example of property-level pasture area and cattle numbers to derive stocking rates, as well as forest cover and deforestation (2013–2017). **b** Bar plots displaying the estimated mean property-level stocking rates in the years 2018–2019, grouped by states, error bars represent standard deviations. **c** Coefficient plots from a state-fixed-effect regression on property-level stocking rates (2018–2019), controlling for local variations. Both (i) deforestation for pasture and (ii) distance to the nearest federal slaughterhouse are significantly negatively correlated with stocking rates. Error bars represent 95% confidence intervals. Image: Google ©2025 CNES/Airbus, Maxar Technologies.

We assessed property-level stocking rates ${{SR}}_{a}$ by integrating cattle densities with property boundaries and corresponding pasture areas (Fig. 3a), and aggregated these to derive state-level averages (Fig. 3b). The overall property-level stocking rate ${{SR}}_{a}$ is 0.73 ± 0.62 cattle per hectare of pasture across states (mean ± standard deviation). Acre exhibits the highest stocking rate (0.85 ± 0.72), while Pará shows the lowest (0.49 ± 0.38). Stocking rates in Amazonas and Rondônia are 0.75 ± 0.71 and 0.72 ± 0.58 cattle per hectare, respectively. These estimates likely represent a lower bound. To better understand these differences, we compared municipal aggregates of our stocking rates with the cattle herd numbers from the annually released municipal livestock survey (Portuguese: Pesquisa Pecuária Municipal, PPM)^53^, and found a moderate positive correlation (r = 0.56, Supplementary Fig. 3, Supplementary Table S1).

To further investigate land management, we integrated moderate and severe pasture degradation and crop plantations maps into our selected cattle-producing properties (Supplementary Fig. 4a–f). Moderate pasture degradation was widespread, affecting 79.4% of the properties, while severe degradation was observed on 19.7% of the properties. Among the states, Pará showed the most extensive median levels of both moderate (47.0% of property area) and severe pasture degradation (8.5% of property area) (Supplementary Fig. 4d–f).

Additionally, little cropland was identified on cattle-producing properties. Our findings indicate the presence of crop plantations on only 0.4% of cattle-producing properties, covering a median area of 4.5%, with only 5 properties in Pará and 7 properties in Rondônia (Supplementary Fig. 4e), a more specific estimate than what previous studies report in broader terms^54,55^.

### Lower stocking rates are linked to past deforestation and lower market access

We estimate linear regression models with state fixed effects to examine the association between deforestation and stocking rates, and between market access and stocking rates. To account for local variations, the regression includes controls for biophysical (temperature and precipitation), socio-economic (population density, baseline stocking rates), and property-level factors (size and forest cover).

We identified a robust negative association between recent deforestation for pasture (2013–2017) and stocking rates in 2018–2019 (${\beta }_{1}\,$= −0.009, *p* < 0.01, Fig. 3c–i). This relationship is notably time-dependent: deforestation occurring closer to the cattle observation period (2016–2017) is associated with significantly lower stocking rates, whereas the effect progressively weakens for deforestation further in the past (Supplementary Tables S2–S5). By 2013, deforestation is no longer significantly correlated with 2018–2019 stocking rates. Although correlative, this temporal pattern suggests a lag between forest-to-pasture conversion and herd buildup, during which newly established pastures sustain fewer cattle. Such delays may reflect land-use strategies oriented toward land occupation or speculation rather than immediate productive use^3,45^.

We further examined the role of market access by relating stocking rates with distance to the nearest federally inspected slaughterhouse (${\beta }_{2}\,$= −0.013, *p* < 0.01, Fig. 3c-ii). Distance to slaughterhouses is significantly and negatively associated with stocking rates, indicating that properties located farther from market infrastructure tend to sustain lower cattle densities. This pattern is consistent with the hypothesis that proximity to markets reduces transaction costs and supports a higher cattle density^46,56^. The stability of both of these relationships was confirmed through a series of robustness checks, including the use of alternative error structures, fixed-effect specifications, and regressions on raw stocking rates without the scale factor (Supplementary Tables S4, S5).

## Discussion

This study introduces a novel approach to analyzing cattle density and derived stocking rates in the Brazilian Amazon by applying a CSR-based density estimation model to VHR satellite imagery. Our method complements existing work that relies on census^13,15^, supply chain^30^, or field survey data^57^ by providing a means to monitor cattle production and land-use change at unprecedentedly fine scales. In the following sections, we evaluate the potential and inherent limitations of this deep learning approach. Furthermore, we assess the implications of satellite-derived cattle density estimation for future land-use research and the development of sustainable agricultural systems in the Amazonian context.

### A novel method to assess livestock production: contribution, limitations, and versatility

By demonstrating a cattle density estimation model in the Brazilian Amazon, we contribute to an emerging field of animal detection in satellite imagery^18^. For the first time to our knowledge, we experimentally validated cattle visibility in VHR images using field-based data and found a 77% detection rate, reflecting that a subset of animals may be obscured by vegetation or farm structure. We corrected this underestimation using a scaling factor of 1.3 in subsequent analyses. The CSR-based model achieved an MAE of 0.30 cattle per hectare, an ensemble-based uncertainty measure, and good generalizability across the four different Amazonian states. Other density-based cattle counting methods show similar performance on small-scale assessments in the Amazon^26^, while object detectors or segmentation-based models are used in other contexts^58,59^. By making model parameters and code publicly available, we aim to support further refinement and adaptation of our methodology.

Predicting cattle density over 12,000 km^2^ in four Amazonian states, our cattle maps complement other existing data on herd size and stocking rates in the region, which are important tools for monitoring production intensity and its environmental impacts. Reported numbers for herd size in Brazil vary by source and methodology: in 2017, the agricultural census recorded 173 million heads, while annual IBGE municipal livestock survey (PPM) estimates from the same year reported 215 million^15,53^. The discrepancy of around 20% is sometimes attributed to undercounting of cattle in the census, as portions of production remain informal^60^. With our scaled stocking rate of 0.73 animals per hectare, our results most likely are below both of these official numbers, as stocking rates derived from census data average 0.91 AU^34^. We note that AU rely on herd composition metrics such as age and weight of the animals; however, they are comparable with simpler cattle head estimations. Further research with greater spatial and temporal coverage is needed to more accurately assess the extent to which our estimates correspond to national statistics.

A key advantage of our method is its ability to identify informal cattle production, allowing for high-resolution spatial understanding of cattle presence, enabling explicit linkage to compliance and governance frameworks, as well as cattle laundering and deforestation leakage^61,62^. Supply chain traceability is a key issue in the Amazonian cattle sector, and Brazil’s largest slaughterhouses have begun implementing traceability and deforestation-free practices^30^. By independently mapping cattle locations, we reveal new opportunities to validate, interpret, and complement existing animal flow data used in the sector^63^. Notably, 13% of detected cattle in our sample are located outside property boundaries, indicating that some production might be unmonitored by environmental policy mechanisms.

Although these findings are particularly relevant for the Amazon, the approach itself is applicable in other contexts. With grasslands comprising roughly 40% of the Earth’s surface^64^ and livestock contributing more than 15% of global GHG emissions^65^, the ability to monitor livestock production at scale represents an emerging frontier in sustainability science. Our approach is versatile and can be applied to other cattle-producing landscapes, including in regions where conventional data are even less available or reliable, such as in South America and Sub-Saharan Africa. However, we note that the Brazilian Amazon provides particularly favorable conditions for further developing and testing this approach. Cattle ranching systems are extensive and characterized by relatively low tree cover on pastures, compared to many livestock production landscapes in Sub-Saharan Africa, where pasture tree densities often exceed 8 trees per hectare^66^.

Despite its broad applicability, our approach has several limitations. Cattle in VHR satellite imagery occupy only a few pixels, making detection inherently difficult and sensitive to image quality. While drone-based studies on cattle detection report much higher accuracies^67^, satellite-based monitoring sacrifices ultra-high spatial resolution in exchange for scalability, enabling consistent observation across large regions. However, the availability of publicly accessible VHR imagery remains very low, commercial acquisition is costly, and imagery licensing restrictions hinder data sharing and scientific reproducibility^67^. Coverage is spatially incomplete and temporally discontinuous, relying on snapshots in time rather than wall-to-wall or time-series data. Finally, image availability in the Amazon is strongly biased toward dry, cloud-free periods, when pasture productivity and cattle densities are typically lower^68^, possibly contributing to our lower-bound stocking rate estimates.

### Towards high-resolution, imagery-based land use models

Unlike most prior work relying on municipality- or landscape-level data^56^, our property-level analysis provides a finer-grained understanding of land-use heterogeneity. Our findings highlight the persistently low stocking rates across the Amazon and widespread extensive cattle ranching (Fig. 3), with minimal crop-livestock integration and prevalent pasture degradation (Supplementary Fig. 4d–f). This aligns with the established literature describing inefficient pasture use in the Amazon^34,37,44,56^ and underscores substantial room for sustainable intensification without further land expansion. However, despite decades of research and policy support^35,55^, the adoption of these practices remains low. High initial investment requirements and weak information transfer to producers are often cited to constrain the uptake of more efficient cattle production systems^69–71^.

Through the first regional-scale mapping of individual cattle across the Brazilian Amazon, we provide more nuanced evidence of the driving mechanisms linking observed livestock densities to deforestation histories and market connectivity. Regression results reveal a negative association between stocking rates and deforestation in preceding years, suggesting that herd build-up after forest clearing is slower than expected under a soil fertility or a boom-and-bust model^42^. As recent syntheses indicate, the links between forest loss and agricultural production are more complex than simple commodity expansion alone^3,31^. Herd expansion may be delayed by financial constraints or broader land speculation cycles^72^. Cattle can also serve as a low-cost strategy for occupying newly deforested land, with the expectation of land appreciation and eventual sale to more capitalized agricultural producers^45^.

We also find a significant negative relationship between distance to the nearest federal slaughterhouse and stocking rates. This result is consistent with the understanding that improved formal market access facilitates intensification and aligns with studies at higher spatial scales^56^. At the same time, the relationship may be more complex than our analysis captures. Federal slaughterhouses represent only one segment of cattle markets; informal and regional slaughtering channels may also influence stocking decisions, but remain poorly documented^73^.

### The path to sustainable agricultural systems in the Amazon

Extensive ranching, deforestation, fire, and degradation of the Amazon forest continue to persist, along with many incremental shifts in monetary incentives for conventional agricultural activities^74^. The need to find ways out of the extensive cattle ranching lock-in is more urgent than ever^75,76^. Decoupling production and deforestation in the Amazon has been difficult for decades, with historical and structural drivers deeply entrenched^44^. While intensification is often promoted as a pathway to reduce pressure on forests, it also carries risks. Without effective governance, productivity gains can trigger rebound or leakage effects, simultaneously increasing production on established properties while incentivizing the opening of new deforestation frontiers^46,77^.

In this context, technology-assisted monitoring can play a critical enabling role^78^. When implemented in a just and equitable manner, fine-scale monitoring can support more transparent governance, inform integrated land-use policies, and help align incentives across actors^79^. Crucially, such tools must be embedded within participatory approaches that support the co-design of production strategies with local producers and policy-makers. Only through the integration of technological capacity with inclusive governance can transitions toward more sustainable land-use systems in the Amazon become viable^80^.

The newest set of policies adopted by Brazil shows promise in promoting more sustainable production systems in the Amazon, aiming to stop deforestation by 2030^28^. Their revised federal action plan includes efforts to initiate a paradigm shift towards more integrated systems, a pillar of the action plan that has been neglected in previous cycles^28^. Yet, international support will be needed to support Brazil in this effort, both by ceasing financial flows to harmful activities like pasture expansion and redirecting them to more valuable and conservation-compatible activities.

## Materials and methods

### VHR satellite imagery

We acquired VHR satellite imagery with RGB (Red, Green, and Blue) bands for the years 2017-2021 and manually annotated the provider (Maxar Technologies and CNES/Airbus) and acquisition date for different large image patches using Google Earth^81^. Images originate from different VHR imagery sensors, including WorldView-3 (ground sampling distance, GSD = 0.30 m), WorldView-2 (GSD = 0.46 m), and Airbus Pleiades (GSD = 0.50 m). We used Google Earth historical imagery to verify individual cattle and match the time range of the field-based cattle numbers. All images were downloaded with the same zoom level corresponding to a ground sampling distance of 0.30 m.

### Cattle verification on VHR satellite imagery

Using field-based cattle counts, we verified the visibility of cattle in five satellite images spanning five municipalities in Acre (Supplementary Data S1). Field-based, property-level cattle numbers were obtained from two sources: (1) semi-annual property-level stock data for 21 farms from the Instituto de Defesa Agropecuária e Florestal (IDAF) database, combined with inflow and outflow transaction records to align with the date of each satellite image; and (2) survey data from 8 additional farms collected in late 2021^47^. Geo-referenced farm locations from the field-based survey were compared with property boundaries obtained from the CAR. The selection of these specific farms prioritized diversity in satellite sensors, imagery acquisition dates, and seasonal coverage. Detection performance was consistent across sensors, with Airbus imagery yielding an 80% detection rate and Maxar imagery 75% (Supplementary Data S1). Due to the limited availability of field data, stratification by additional farm characteristics remained unfeasible, and the validation could not be extended to other regions. Although we use the detection rate to derive a general scale factor for the CSR-based cattle density estimations, we note that the representativeness of the field-based data set might not cover all production system heterogeneity, region- and site-specific vegetation conditions. However, we note that similar extensive cattle ranching practices and tree cover are expected across the assessed region^40^.

### Generation of training and test sets

Training data selection followed a stratified approach to mitigate sensor-specific artifacts and environmental variability. In doing so, we integrated imagery from distinct providers (Maxar and CNES/Airbus) while spatial and environmental diversity was manually ensured by sampling from five geographically distinct municipalities in the state of Acre and the state of Pará. Images were processed using a tiling strategy into patches ranging from 400 × 400 to 420 × 420 pixels, corresponding to a ground sampling of ~120 m × 120 m. Bounding boxes around individual animals were drawn in LabelImg^82^ and then converted into density maps using a Gaussian filter with a kernel size of 5^25^. The training and validation set consists of 6284 patches with cattle herds containing 18,400 labeled cattle. We note that, as more than 80% of the cattle stocks in the Amazon are of the Nelore *Bos indicus* breed^83^, the majority of the training data set represents low-pigmented (i.e., white) cattle; the exact share of white cattle has not been assessed.

For the test set, to ensure geographic representativeness across the Brazilian Amazon, we employed a stratified sampling approach targeting all covered states, selecting tiles that represent a gradient of cattle densities. For each state, at least two distinct imagery sources were manually selected to account for within-state pasture heterogeneity and differences in satellite sensors, resulting in 495 image tiles with a total of 639 cattle. The training set was annotated by the first author and validated by 10 different verification assistants. The test set was constructed by the first author, with data annotation performed by a labeling assistant and subsequent quality assurance conducted by a second verification assistant.

### Model architecture, training, and testing

To predict cattle numbers, we used a CNN-based density estimation model, based on the congested scene recognition (CSR) architecture as described in Li et al.^25^, a method used before to detect cattle^26^. Our model instance is designed with a VGG-16^84^ backbone for feature extraction with 3 ×3 kernels for a constant receptive field size and trained on the ImageNet data set. The decoder includes dilation, sparsely enlarging receptive fields by a dilation factor of 2, leading to 5 ×5 kernels, but without increasing the number of parameters. The model output, consisting of a density map, is 8 times smaller than the image input size. We utilize a Mean Squared Error loss function and Adam optimizer with a fixed learning rate of 1 × 10^−5^ to train the model. The network was trained in 100,000 iterations, with a batch size of 16 and horizontal flipping for data augmentation. We used Python 3.9 and PyTorch 1.13.1^85^ to implement the CSRNet architecture.

To test the model, we compared the predicted cattle numbers of the model outputs to the manually labeled cattle in the test set patches C_i_^GT^, indexed by i = 1, …, N. In the case of the CSR model, the predicted number of animals C_i_ for patch i is the sum of the pixel values of the model output density map, i.e., C_i_ = ${\sum }_{w=1}^{W/8}{\sum }_{l=1}^{L/8}{z}_{l,w,i}$. L, W are the length and width of the image, and z_l, w, i_ is the pixel value at (l,w) of the density map (which is L/8 x W/8 in size). The error rate was calculated as the mean absolute error (MAE) between Ci and Ci^GT^ for all N test patches. This error was then converted into units of cattle per hectare by considering the GSD of 0.3 and the size of each input image patch (set to 400 px x 400 px, corresponding to 120 m × 120 m).

$${{MAE}}_{[{cattle}/{hectare}]}=\,0.694\,\times \frac{1}{N}{\sum }_{i=1}^{N}\left|{C}_{i}-\,{C}_{i}^{{GT}}\right|$$

Additional test set performance metrics, including mean absolute percentage error and mean error for stratified samples across cattle-density classes and Brazilian states, as well as example model outputs on Maxar and Airbus imagery, are presented in Supplementary Figs. S1, S2.

### Ensemble method to measure uncertainty

We noted variations in model performance across different images, which were largely due to differences in image quality and the contrast between cattle and their background. To address this, our model not only provides predictions but also estimates an uncertainty value to flag low-confidence predictions. We use an ensemble of five models with randomly initialized network parameters. For each target pixel, we calculated the mean (z_p_) and standard deviation (σ_p_) from the single model outputs, which resulted in the cattle number and uncertainty measure (as in Fig. 1b), respectively. This ensemble approach enables a more robust prediction through model averaging and the estimation of epistemic uncertainty. The discrepancy between predictions is typically higher on samples corresponding to challenging parts of the data distribution. Hence, the standard deviation of the predictions σ_p_, helps measure the reliability of the prediction for a given image^86^.

### Prediction on the full imagery set

The ensemble CSRNet was applied to 170 satellite image patches with an average of 71.2 km^2^ and a total of 12,260 km^2^. Satellite images were selected based on availability until saturation. Full details on image outlines, including acquisition dates, data provider (140 images from Maxar, 30 images from Airbus), and geo-referencing information, are provided in Supplementary Data S2. For all stocking rate estimations (Fig. 3b, Supplementary Fig. 3), land use characterization (Supplementary Fig. 4a–f), and regression analysis (Fig. 3c), we used imagery of the years 2018-2019, the years with the highest abundance of VHR images at the time of data acquisition. We combined the two years to have higher spatial coverage.

We created cattle densities consisting of geo-referenced data points in the center of each patch, detailing both the estimated cattle numbers μ_a_ and the ensemble standard deviation σ (or model uncertainty) of these estimates (Supplementary Data S3). The sum of the cattle was rounded to whole numbers.

### Stocking rate estimations and secondary data integration

#### Overall stocking rates (${SR}$)

Using pasture maps from MapBiomas^27^, we derive the stocking rates by using CSR-based cattle numbers divided by the mean pasture area within VHR imagery outlines and scaled with the detection rate-derived factor of 1.3.

#### Stocking rates at the property-level

Next, we used the CAR to aggregate cattle numbers at the property level. For each property a with an image patch set P_a_ within the image borders, we summed up the ensemble mean μ_a_ of the predicted cattle number within the property and added a scale factor of 1.3 (see also Supplementary Fig. 4a). We calculated the mean pasture area ${{Past}}_{a}$ for the years 2018 and 2019. Property stocking rates (SR_a_) were then calculated as

$${{SR}}_{a}=\frac{1.3* {\sum }_{{P}_{a}}{\mu }_{a}}{{{Past}}_{a}}$$

Out of 7701 property boundaries from the CAR that were fully covered by the image outlines, 60% showed cattle (we filtered for a minimum of five animals to reduce the influence of detection noise). We focused on properties between 5 and 800 hectare in total area to exclude very small holdings with irregular land-use patterns and very large properties that may be managed heterogeneously. Further, to mitigate the influence of outliers, we removed the 10% of property-level estimates with the highest mean uncertainty σ (a threshold empirically determined on validation data).

#### Municipal stocking rates

We derived stocking rates on the municipality level by using the cattle mapping-derived cattle numbers per municipality (scaled by 1.3) and mean pasture area within VHR imagery outlines (Supplementary Fig. 3a). We filtered for municipalities for which we have imagery for at least 50 km^2^ pasture area, representing a cutoff below which sampling variability increased sharply, and estimates became unstable. Supplementary Table S1 further provides a summary of the proportion of pasture captured.

We then compared our municipal stocking rates with official estimates, derived from the municipal livestock survey (PPM), averaging animal head numbers for 2018–2019 and dividing by the mean municipal pasture area over the same years, following conventions in the literature^87^. The Pearson correlation between our mean municipal stocking rates and official estimates was calculated using the cor() function in R (Supplementary Fig. 3b).

#### Other land uses

Mean crop area for 2018 and 2019 was derived from the MapBiomas classes ‘mosaic of crops’ and ‘soybeans’, while other crop classes were not represented in the sample. Similarly, mean pasture degradation for moderate degradation and severe degradation classes, as defined by MapBiomas^27^, was calculated for each property (Supplementary Fig. 4). Both mean crop area and pasture degradation showed no correlation with stocking rates.

### Regression analysis

We employed a state fixed-effects (FE) regression model to account for state-specific characteristics and added control variables to account for local variations, allowing us to assess the association of different variables with the stocking rate (SRₐ) for each property a.

We included ${{Deforestation}}_{a,\left[t-6,t-2\right]}$ as the proportion of property a’s area that was deforested and converted into pasture in a four-year window preceding the observation period. This lag structure allows us to focus on the medium-term association of forest clearing with herd development. We selected 2013 as the baseline year because interactions between deforestation and cattle stocking rates are expected to materialize within a medium-term window governed by biological herd dynamics and pasture establishment processes^88^. Deforestation for other land uses, such as crop plantations, is negligible in our study region (Supplementary Fig. 4)^2^.

We further assumed that higher market access would positively influence stocking rates. To measure market access, we used a georeferenced slaughterhouse data set from the Transparency for Sustainable Economies (Trase) initiative^89^ to calculate the distance to the closest slaughterhouse $({{DistSlaughterhouse}}_{a})$.

To account for local variations, we included several controls into our model. ${{PropertySize}}_{a}$ was used to control for differences in landowner classes, which determines resource access, such as credits for farm investments, commonly used in the literature as a control variable^16,90^. *PopulationDensity*_*a*_^91^ at the municipality level was included in the model to control for the development rate in the region. We further included a baseline$\,{{PPMStockingRates}( \% )}_{a,t-6}$, derived from the municipal livestock survey (PPM) of the year 2013 and pasture area for the year 2013 at the municipality level to account for pre-existing differences in production intensity across regions. ${{ForestCover}( \% )}_{a,t-6}$, measured as the share of forest cover in 2013 at the property level, was incorporated to represent baseline forest availability, while ${{ForestCover\_Buffer}}_{a,t-6\,}$ represents forest cover in a 10 km buffer region around the property to account for regional forest availability.

AvgPrecipitation_a_ and AvgTemperature_a_ are included as biophysical control factors influencing pasture growth and, therefore, stocking rate capacity^34^. For precipitation, we use the NASA Monthly Global Precipitation Measurement (GPM) v6 with a resolution of 27,830 m^92^, and for temperature, we use the measurements from the Global Change Observation Mission with a resolution of 4638 m^93^, both taking the mean over 2018–2019 for each municipality. The data used are further described in Supplementary Table S2.

The FE-Full includes the following estimators:

$${{SR}}_{a,t}= 	{\beta }_{1}{{Deforestation}}_{a,\left[t-6,t-2\right]}+{\beta }_{2}{{DistSlaughterhouse}}_{a,t}\\ 	 {+\beta }_{3}{Property}{{Size}}_{a,t}+{\beta }_{4}{{PPMStockingRate}}_{a,t-6\,}\\ 	 +{\beta }_{5}{{ForestCover}{{\rm{\_}}}{Buffer}}_{a,t-6\,}+{\beta }_{6}{{ForestCover}}_{a,t-6\,}\\ 	 +{\beta }_{7}{{AvgPrecipitation}}_{a,t}+{\beta }_{8}{{AvgTemperature}}_{a,t}\\ 	 +{{\beta }_{9}{PopulationDensity}}_{a,t}+{\delta }_{s}+{\epsilon }_{a},$$

where $\epsilon$_a_ is the error term, and δ_s_ is the state fixed effects. Errors were clustered at the municipality level. The summary statistics of the variables are available in Supplementary Table S3. In Supplementary Table S4, full model parameters include fixed effects with clustered errors, as well as heteroskedasticity-robust errors and a model specification with single-year deforestation variables for 2013–2017. Furthermore, we present the estimates of the regression for unscaled cattle stocking density in Supplementary Table S5. We show the fixed-effects model with errors clustered at the municipality level in our main results because it is the most conservative model. Confidence intervals for Fig. 3 were calculated following Figueiras et al.^94^.

R^95^, the R stats packages for statistical tests, geoprocessing packages sf^96^ (v. 1.0.19) and exactextractr^97^ (v. 0.10.0) were used for spatial analysis. The buffer metrics were calculated using standard geospatial functions in the sf package. The package fixest^98^ (v. 0.12.1) was used for regression models.

## Supplementary information

Peer review file

Supplementary Information

Description of Additional Supplementary Files

Supplementary Data S2

Supplementary Data S1

Supplementary Data S3

## Data Availability

Details of datasets for the analysis and download links are provided in the Supplementary Information. Please note that the underlying very-high-resolution (VHR) satellite imagery used for training and inference cannot be shared publicly due to third-party copyright restrictions. However, raw image footprints, timestamps, and derived cattle density outputs and regression variables are provided in the Supplementary Information.

## Code availability

The CSR model and data set are available on https://github.com/leoniehodel/deepCattleCount, and the pre-trained model parameters are available on 10.5281/zenodo.13385687, Supporting code and data for the regression analysis are accessible via 10.24433/CO.4884776.v2.

## References

[1] Curtis, P. G., Slay, C. M., Harris, N. L., Tyukavina, A. & Hansen, M. C. Classifying drivers of global forest loss. *Science* **361**, 1108–1111 (2018). 30213911 10.1126/science.aau3445

[2] Haddad, E. A. et al. Economic drivers of deforestation in the Brazilian Legal Amazon. *Nat. Sustain.* **7**, 1141–1148 (2024).

[3] Pendrill, F. et al. Disentangling the numbers behind agriculture-driven tropical deforestation. *Science* **377**, eabm9267 (2022). 36074840 10.1126/science.abm9267

[4] Meyfroidt, P., Rudel, T. K. & Lambin, E. F. Forest transitions, trade, and the global displacement of land use. *Proc. Natl. Acad. Sci. USA* **107**, 20917–20922 (2010). 21078977 10.1073/pnas.1014773107 PMC3000287

[5] Garrett, R. D., Levy, S. A., Gollnow, F., Hodel, L. & Rueda, X. Have food supply chain policies improved forest conservation and rural livelihoods? A systematic review. *Environ. Res. Lett.* **16**, 033002 (2021).

[6] Soares-Filho, B. et al. Cracking Brazil’s forest code. *Science* **344**, 363–364 (2014). 24763575 10.1126/science.1246663

[7] Alix-Garcia, J., Rausch, L. L., L’Roe, J., Gibbs, H. K. & Munger, J. Avoided deforestation linked to environmental registration of properties in the Brazilian Amazon. *Conserv. Lett.* **11**, e12414 (2018).

[8] Austin, K. G. et al. Mapping and monitoring zero-deforestation commitments. *BioScience* **71**, 1079–1090 (2021). 34616238 10.1093/biosci/biab082 PMC8490929

[9] Farjon, G., Huijun, L. & Edan, Y. Deep-learning-based counting methods, datasets, and applications in agriculture: a review. *Precis. Agric.* **24**, 1683–1711 (2023).

[10] Tolan, J. et al. Very high-resolution canopy height maps from RGB imagery using self-supervised vision transformer and convolutional decoder trained on aerial lidar. *Remote Sens. Environ.* **300**, 113888 (2024).

[11] Pendrill, F. et al. Agricultural and forestry trade drives large share of tropical deforestation emissions. *Glob. Environ. Change* **56**, 1–10 (2019).

[12] Hayek, M. N., Harwatt, H., Ripple, W. J. & Mueller, N. D. The carbon opportunity cost of animal-sourced food production on land. *Nat. Sustain.* **4**, 21–24 (2021).

[13] Gilbert, M. et al. Global distribution data for cattle, buffaloes, horses, sheep, goats, pigs, chickens and ducks in 2010. *Sci. Data* **5**, 180227 (2018). 30375994 10.1038/sdata.2018.227 PMC6207061

[14] Herrero, M. et al. Biomass use, production, feed efficiencies, and greenhouse gas emissions from global livestock systems. *Proc. Natl. Acad. Sci. USA* **110**, 20888–20893 (2013). 24344273 10.1073/pnas.1308149110 PMC3876224

[15] IBGE. Censo Agropecuário. https://www.ibge.gov.br/estatisticas/economicas/agricultura-e-pecuaria/21814-2017-censo-agropecuario.html?edicao=25757&t=resultados (2017).

[16] Skidmore, M. E. et al. Cattle ranchers and deforestation in the Brazilian Amazon: production, location, and policies. *Glob. Environ. Change* **68**, 102280 (2021).

[17] Azevedo-Ramos, C. et al. Lawless land in no man’s land: the undesignated public forests in the Brazilian Amazon. *Land Use Policy* **99**, 104863 (2020).

[18] Wu, Z. et al. Deep learning enables satellite-based monitoring of large populations of terrestrial mammals across heterogeneous landscape. *Nat. Commun.* **14**, 3072 (2023). 37244940 10.1038/s41467-023-38901-y PMC10224963

[19] Persello, C. et al. Deep learning and Earth observation to support the sustainable development goals: current approaches, open challenges, and future opportunities. *IEEE Geosci. Remote Sens. Mag.* **10**, 172–200 (2022).

[20] Duporge, I., Isupova, O., Reece, S., Macdonald, D. W. & Wang, T. Using very-high-resolution satellite imagery and deep learning to detect and count African elephants in heterogeneous landscapes. *Remote Sens. Ecol. Conserv.* **7**, 369–381 (2021).

[21] Gonçalves, B. C., Spitzbart, B. & Lynch, H. J. SealNet: a fully-automated pack-ice seal detection pipeline for sub-meter satellite imagery. *Remote Sens. Environ.* **239**, 111617 (2020).

[22] Guirado, E., Tabik, S., Rivas, M. L., Alcaraz-Segura, D. & Herrera, F. Whale counting in satellite and aerial images with deep learning. *Sci. Rep.* **9**, 14259 (2019). 31582780 10.1038/s41598-019-50795-9 PMC6776647

[23] Rodríguez, A. C., D’Aronco, S., Schindler, K. & Wegner, J. D. Mapping oil palm density at country scale: an active learning approach. *Remote Sens. Environ.* **261**, 112479 (2021).

[24] Rodriguez, A. C. & Wegner, J. D. Counting the uncountable: deep semantic density estimation from space. In *German Conference on Pattern Recognition*, pp. 351–362. (Springer International Publishing, 2018).

[25] Li, Y., Zhang, X. & Chen, D. CSRNet: dilated convolutional neural networks for understanding the highly congested scenes. in *2018 IEEE/CVF Conference on Computer Vision and Pattern Recognition* 1091–1100 (IEEE, 2018).

[26] Laradji, I. et al. Counting cows: tracking illegal cattle ranching from high-resolution satellite imagery. Preprint at https://arxiv.org/abs/2011.07369 (2020).

[27] MapBiomas. Projeto MapBiomas - Coleção v7 da Série Anual de Mapas de Cobertura e Uso de Solo do Brasil. https://mapbiomas.org/en (2023).

[28] Brasil. Plano de Ação para Prevenção e Controle do Desmatamento na Amazônia Legal (PPCDAm), 5ª fase (2023–2027). Brasília (2023).

[29] Brasil. *National Biodiversity Strategy and Action Plan*. https://www.cbd.int/doc/world/br/br-nbsap-v3-en.pdf (2017).

[30] Levy, S. A., Cammelli, F., Munger, J., Gibbs, H. K. & Garrett, R. D. Deforestation in the Brazilian Amazon could be halved by scaling up the implementation of zero-deforestation cattle commitments. *Glob. Environ. Change* **80**, 102671 (2023).

[31] zu Ermgassen et al. Halting the expansion of pasture in the Brazilian Amazon. *One Earth* **7**, 1923–1926 (2024.

[32] Latawiec, A. E., Strassburg, B. B. N., Valentim, J. F., Ramos, F. & Alves-Pinto, H. N. Intensification of cattle ranching production systems: socioeconomic and environmental synergies and risks in Brazil. *Animal* **8**, 1255–1263 (2014). 26263189 10.1017/S1751731114001566

[33] Telles, T. S. et al. Livestock changes in Brazil and sustainable intensification challenges. *Agronomy* **14**, 2429 (2024).

[34] Strassburg, B. B. N. et al. When enough should be enough: improving the use of current agricultural lands could meet production demands and spare natural habitats in Brazil. *Glob. Environ. Change* **28**, 84–97 (2014).

[35] Gil, J. et al. Tradeoffs in the quest for climate smart agricultural intensification in Mato Grosso, Brazil. *Environ. Res. Lett.* **13**, 064025 (2018).

[36] Cohn, A. S. et al. Cattle ranching intensification in Brazil can reduce global greenhouse gas emissions by sparing land from deforestation. *Proc. Natl. Acad. Sci. USA* **111**, 7236–7241 (2014). 24778243 10.1073/pnas.1307163111 PMC4034253

[37] Arantes, A. E., Couto, V. R., de, M., Sano, E. E. & Ferreira, L. G. Livestock intensification potential in Brazil based on agricultural census and satellite data analysis. *Pesqui. Agropecuária Bras.* **53**, 1053–1060 (2018).

[38] de Souza, L. I., de Carvalho, T. B., Monteiro, C., Biscalchin, R. M. & Domingos, I. Índice de produtividade da pecuária de corte: uma aplicação no TAC da Carne. *Boi Na Linha* (2019).

[39] Valentim, J. & Andrade, C. M. Tendências e perspectivas da pecuária bovina na amazônia brasileira. *Amaz. Ciěncia Desenvolv*. **4**, (2009).

[40] Walker, R. et al. Ranching and the new global range: Amazônia in the 21st century. *Geoforum* **40**, 732–745 (2009).

[41] Ceddia, M. G. The impact of income, land, and wealth inequality on agricultural expansion in Latin America. *Proc. Natl. Acad. Sci. USA* **116**, 2527–2532 (2019). 30679279 10.1073/pnas.1814894116 PMC6377487

[42] Caviglia-Harris, J. et al. Busting the boom–bust pattern of development in the Brazilian Amazon. *World Dev* **79**, 82–96 (2016).

[43] Assunção, J., Gandour, C., Rocha, R. & Rocha, R. The effect of rural credit on deforestation: evidence from the Brazilian Amazon. *Econ. J.* **130**, 290–330 (2020).

[44] Bowman, M. S. et al. Persistence of cattle ranching in the Brazilian Amazon: a spatial analysis of the rationale for beef production. *Land Use Policy* **29**, 558–568 (2012).

[45] Miranda, J., Börner, J., Kalkuhl, M. & Soares-Filho, B. Land speculation and conservation policy leakage in Brazil. *Environ. Res. Lett.* **14**, 045006 (2019).

[46] Santos, A. B. & Costa, M. H. Do large slaughterhouses promote sustainable intensification of cattle ranching in Amazonia and the Cerrado? *Sustainability* **10**, 3266 (2018).

[47] Hodel, L. *Cattle, Culture, and Feminist Ecologies in the Brazilian Amazon: Advances in Theoretical and AI-Driven Land System Science* (ETH Zurich, 2023).

[48] Andrade, C. M. S. de, Salman, A. K. D. & Oliveira, T. K. de. Arborização de Pastagens Na América Latina. In *Guia ARBOPASTO: manual de identificação e seleção de espécies arbóreas para sistemas silvipastoris* (eds Andrade, C. M. S. de, Salman, A. K. D. & Oliveira, T. K. de) (Brasília, DF: Embrapa, 2012).

[49] Andrade, C. M. S. de & Macedo, V. H. M. Pecuária de cria no Acre: Infraestrutura produtiva e gestão da propriedade. *Embrapa Acre*. http://www.infoteca.cnptia.embrapa.br/infoteca/handle/doc/1169943 (2024).

[50] Barrett, K., Valentim, J. & Turner, B. L. Ecosystem services from converted land: the importance of tree cover in Amazonian pastures. *Urban Ecosyst* **16**, 573–591 (2013).

[51] Carvalho, R., de Aguiar, A. P. D. & Amaral, S. Diversity of cattle raising systems and its effects over forest regrowth in a core region of cattle production in the Brazilian Amazon. *Reg. Environ. Change* **20**, 44 (2020).

[52] Sistema Nacional de Cadastro Ambiental Rural (Sicar). https://www.car.gov.br/ (2022).

[53] IBGE. Pesquisa da Pecuária Municipal. https://www.ibge.gov.br/estatisticas/economicas/agricultura-e-pecuaria/9107-producao-da-pecuaria-municipal.html (2024).

[54] Gianetti, G. W., Ferreira Filho, J. B. & de, S. O. Plano e Programa ABC: uma análise da alocação dos recursos. *Rev. Econ. E Sociol. Rural* **59**, e216524 (2021).

[55] Gil, J. D. B., Garrett, R. & Berger, T. Determinants of crop-livestock integration in Brazil: evidence from the household and regional levels. *Land Use Policy* **59**, 557–568 (2016).

[56] Garrett, R. D. et al. Intensification in agriculture-forest frontiers: land use responses to development and conservation policies in Brazil. *Glob. Environ. Change* **53**, 233–243 (2018).

[57] zu Ermgassen, E. K. H. J. et al. Results from on-the-ground efforts to promote sustainable cattle ranching in the Brazilian Amazon. *Sustainability* **10**, 1301 (2018).

[58] Ocholla, I. A. et al. Livestock detection and counting in Kenyan Rangelands using aerial imagery and deep learning techniques. *Remote Sens*. **16**, 2929 (2024).

[59] Robinson, C., Ortiz, A., Hughey, L., Stabach, J. A. & Ferres, J. M. L. Detecting cattle and elk in the wild from space. Preprint at https://arxiv.org/abs/2106.15448 (2021).

[60] Schmitt, C. J. *Rebanhos sustentáveis? Perspectivas e controvérsias em torno da ambientalização da pecuária brasileira*. (Fundação Heinrich Böll, Rio de Janeiro, RJ, 2022).

[61] Alix-Garcia, J. & Gibbs, H. K. Forest conservation effects of Brazil’s zero deforestation cattle agreements undermined by leakage. *Glob. Environ. Change* **47**, 201–217 (2017).

[62] West, T. A. P., Rausch, L., Munger, J. & Gibbs, H. K. Protected areas still used to produce Brazil’s cattle. *Conserv. Lett.* **15**, e12916 (2022).

[63] Choquet, P.-L. Data under siege: making cattle flows (il)legible in the Brazilian Amazon. *Environ. Plan. E Nat. Space* **8**, 1465–1485 (2025).

[64] Parente, L. et al. Annual 30-m maps of global grassland class and extent (2000–2022) based on spatiotemporal Machine Learning. *Sci. Data* **11**, 1303 (2024). 39663386 10.1038/s41597-024-04139-6 PMC11634896

[65] Hayek, M. N. & Miller, S. M. Underestimates of methane from intensively raised animals could undermine goals of sustainable development. *Environ. Res. Lett.* **16**, 063006 (2021).

[66] Esquivel-Mimenza, H., Ibrahim, M., Harvey, C. A., Benjamin, T. & Sinclair, F. Dispersed trees in pasturelands of cattle farms in a tropical dry ecosystem. *CIFOR-ICRAF* https://www.cifor-icraf.org/knowledge/publication/33018/ (2011).

[67] Barbedo, J. G. A., Koenigkan, L. V., Santos, T. T. & Santos, P. M. A study on the detection of cattle in UAV images using deep learning. *Sensors* **19**, 5436 (2019). 31835487 10.3390/s19245436 PMC6960676

[68] Skidmore, M. E. Outsourcing the dry season: Cattle ranchers’ responses to weather shocks in the Brazilian Amazon. *Am. J. Agric. Econ.* **105**, 409–433 (2023).

[69] Bragança, A. et al. Extension services can promote pasture restoration: Evidence from Brazil’s low carbon agriculture plan. *Proc. Natl. Acad. Sci. USA* **119**, e2114913119 (2022). 35298338 10.1073/pnas.2114913119 PMC8944583

[70] Garrett, R. et al. Explaining the persistence of low income and environmentally degrading land uses in the Brazilian Amazon. *Ecol. Soc*. **22**, 6270164 (2017).

[71] Hodel, L., le Polain de Waroux, Y. & Garrett, R. D. Characterizing culture’s influence in land systems. *Nat. Sustain*. 1–10 (2024).

[72] Fearnside, P. M. Deforestation in Brazilian Amazonia: history, rates, and consequences. *Conserv. Biol.* **19**, 680–688 (2005).

[73] Brandão Jr, A., Rausch, L., Munger, J. & Gibbs, H. K. Mapping slaughterhouse supply zones in the Brazilian Amazon with cattle transit records. *Land* **12**, 1782 (2023).

[74] Merry, F. & Soares-Filho, B. Will intensification of beef production deliver conservation outcomes in the Brazilian Amazon? *Elem. Sci. Anthr.* **5**, 24 (2017).

[75] Abramovay, R. et al. Chapter 30: Opportunities and challenges for a healthy standing forest and flowing rivers bioeconomy in the Amazon. In *Amazon Assessment Report 2021* (eds Nobre, C. et al.) (UN Sustainable Development Solutions Network (SDSN), 2021).

[76] Flores, B. M. et al. Critical transitions in the Amazon forest system. *Nature* **626**, 555–564 (2024). 38356065 10.1038/s41586-023-06970-0 PMC10866695

[77] Goulart, F. F., Chappell, M. J., Mertens, F. & Soares-Filho, B. Sparing or expanding? The effects of agricultural yields on farm expansion and deforestation in the tropics. *Biodivers. Conserv.* **32**, 1089–1104 (2023).

[78] Camilleri, M. A. Artificial intelligence governance: ethical considerations and implications for social responsibility. *Expert Syst* **41**, e13406 (2024).

[79] Grabs, J., Cammelli, F., Levy, S. A. & Garrett, R. D. Designing effective and equitable zero-deforestation supply chain policies. *Glob. Environ. Change* **70**, 102357 (2021).

[80] Camaréna, S. Artificial intelligence in the design of the transitions to sustainable food systems. *J. Clean. Prod.* **271**, 122574 (2020).

[81] Google Earth. https://earth.google.com (2026).

[82] Tzutalin, D. LabelImg. https://github.com/HumanSignal/labelImg (2015).

[83] Piccoli, M. L. et al. Origins and genetic diversity of British cattle breeds in Brazil assessed by pedigree analyses. *J. Anim. Sci.* **92**, 1920–1930 (2014). 24671583 10.2527/jas.2013-7283

[84] Simonyan, K. & Zisserman, A. Very Deep Convolutional Networks for Large-Scale Image Recognition. Preprint at https://arxiv.org/abs/1409.1556 (2015).

[85] Paszke, A. et al. PyTorch: An Imperative Style, High-Performance Deep Learning Library. in *Advances in Neural Information Processing Systems* vol. 32 (NIPS, 2019).

[86] Kendall, A. & Gal, Y. What uncertainties do we need in Bayesian deep learning for computer vision? Preprint at https://arxiv.org/abs/1703.04977 (2017).

[87] Allen, V. G. et al. An international terminology for grazing lands and grazing animals. *Grass Forage Sci* **66**, 2–28 (2011).

[88] Fernandes, E. C., Rosa, B. L., Valentim, J. F., Gomes, F. C. D. R. & Lambertucci, D. M. Dinâmica de indicadores da pecuária bovina de corte na Amazônia: evidências do estado do Acre (2013–2024). *Rev. Econ. E Sociol. Rural* **63**, e295238 (2025).

[89] Trase. Trase cattle logistics map v2.0. trase.earth (2021).

[90] L’Roe, J., Rausch, L., Munger, J. & Gibbs, H. K. Mapping properties to monitor forests: Landholder response to a large environmental registration program in the Brazilian Amazon. *Land Use Policy* **57**, 193–203 (2016).

[91] IBGE. Estimativas da população residente para os municípios e para as unidades da federação. https://www.ibge.gov.br/estatisticas/sociais/populacao/9103-estimativas-de-populacao.html (2023).

[92] Huffman, G. J. “GPM IMERG final precipitation L3 half hourly 0.1 degree× 0.1 degree V06.” https://developers.google.com/earth-engine/datasets/catalog/NASA_GPM_L3_IMERG_V06 (2019).

[93] Moriyama, M. GCOM–C1/SGLI Land Surface Temperature Product Algorithm Theoretical Basis Document (v2) https://developers.google.com/earth-engine/datasets/catalog/JAXA_GCOM-C_L3_LAND_LST_V2 (2020).

[94] Figueiras, A., Domenech-Massons, J. M. & Cadarso, C. Regression models: calculating the confidence interval of effects in the presence of interactions. *Stat. Med.* **17**, 2099–2105 (1998). 9789916 10.1002/(sici)1097-0258(19980930)17:18<2099::aid-sim905>3.0.co;2-6

[95] R Core Team. *R: A Language and Environment for Statistical Computing* (R Core Team, 2022).

[96] Pebesma, E. et al. sf: Simple Features for R. 10.32614/CRAN.package.sf (2023).

[97] Baston, D., ISciences & LLC. exactextractr: Fast Extraction from Raster Datasets using Polygons. 10.32614/CRAN.package.exactextractr (2023).

[98] Berge, L., Krantz, S., McDermott, G. & Lenth, R. fixest: Fast Fixed-Effects Estimations 10.32614/CRAN.package.fixest (2024).

