# Supplementary material for: Spatial patterns of cattle densities across the Brazilian Amazon revealed by very high-resolution satellite imagery
Source: Commun Sustain. 2026 Jun 16;1(1):98. doi: 10.1038/s44458-026-00082-2 (PMC13271885; doi:10.1038/s44458-026-00082-2)
Supplement: Supplementary file 2 — Supplementary Information [file 44458_2026_82_MOESM2_ESM.pdf]

Supplementary Information for the manuscript

Spatial patterns of cattle densities across the Brazilian Amazon revealed by very high-resolution satellite imagery

Leonie Hodel et al., lh837@cam.ac.uk

This file includes:

Supplementary Figures S1-S4
Supplementary Tables S1-S5
Supplementary Data S1, S2 in separate Excel files
Supplementary Data S3 in a separate GeoJSON file

Supplementary Figure S1.

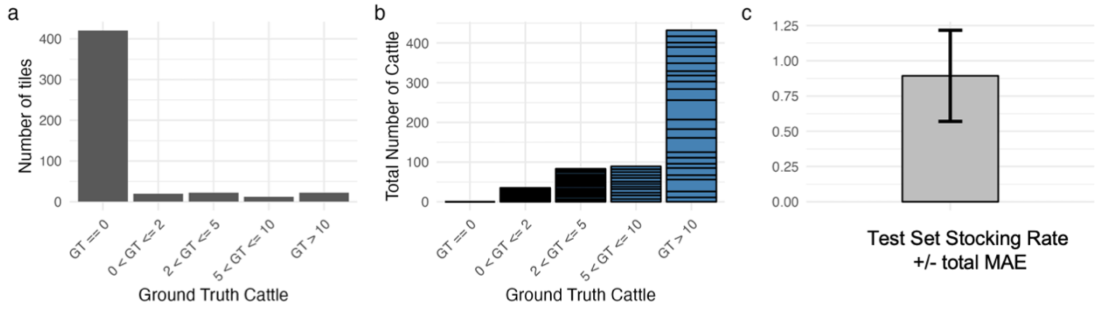

$$MAE = \frac{1}{N}\sum_{i=1}^{N}\left|C_i - C_i^{GT}\right|,\ MAPE = \frac{1}{N}\sum_{i=1}^{N}\frac{\left|C_i - C_i^{GT}\right|}{\{C_i^{GT},1\}},\ ME = \frac{1}{N}\sum_{i=1}^{N} C_i^{GT} - C_i\ .$$

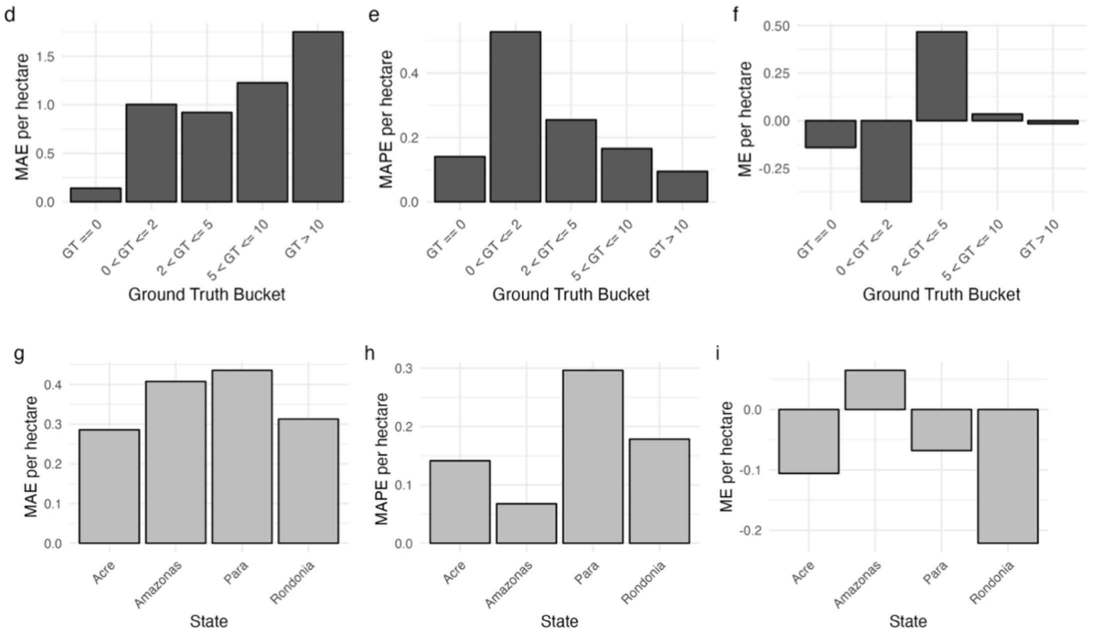

Supplementary Figure S1: Test set characteristics and outputs for the Congested Scene Recognition model, consisting of a total of 495 very high-resolution satellite image tiles with a total of 639 cattle (GT = Ground truth represents manually labeled cattle on images). (a) Test set tiles for different cattle densities. Most of the tiles in the test set do not depict any cattle, reflecting the sparsity of cattle in the landscape. (b) Total number of cattle for different cattle densities in the test set. (c) Overall model performance: the ground truth stocking rate of the test set is 0.89 cattle per hectare, and the mean absolute error (MAE) is 0.30 cattle per hectare. (d-f) MAE, mean absolute percentage error (MAPE) and mean error (ME) for different densities of GT cattle numbers. The ME indicates that the model tends to overcount in the low-cattle density bin (0<GT<=2, negative ME) and to undercount in the medium density bin (2<GT<=5, positive ME). (g-i) MAE, MAPE, and ME for four different states in the Brazilian Amazon. Images from Acre show the lowest MAE, while those from Pará have the highest MAE. Predictions in the state of Amazonas show a slight positive ME, while predictions in Rondônia show a negative ME.

Supplementary Figure S2.

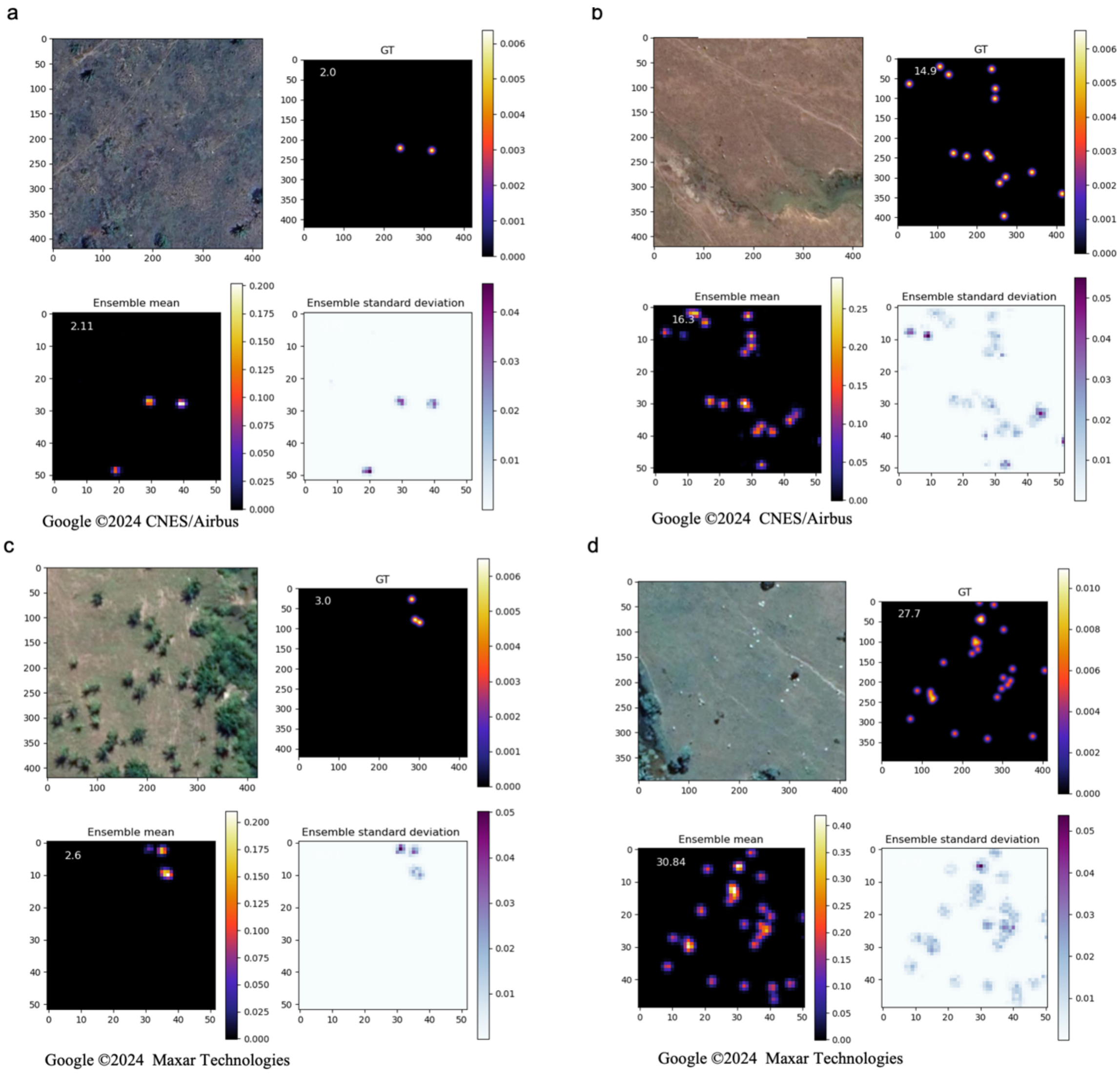

Supplementary Figure S2: Example image tiles of 400-420 pixels and ground truth (GT) labels, ensemble mean predictions and model uncertainty (ensemble standard deviation) of the Congested Scene Recognition model (a-b) Airbus images with (a) low and (b) high cattle numbers, (c-d) Maxar images with (c) low and (d) high cattle numbers.

Supplementary Table S1.

Variable	N	Mean	Std. Dev.	Min	Pctl. 25	Pctl. 75	Max
Municipality	47						
... Acre	17	36%					
... Amazonas	5	11%					
... Pará	12	26%					
... Rondônia	13	28%					
Total municipal pasture area (km^2)	47	2206	1849	270	1198	2406	8564
Cattle count from PPM	47	290957	213885	16582	159990	350765	927584
Stocking rate	47	1.4	0.56	0.29	0.95	1.8	2.5
Pasture observed in VHR images (km^2)	47	153	101	56	82	184	537
Cattle count from density maps	47	7540	7970	146	3068	8882	47891
Scaled Stocking rate	47	0.62	0.3	0.033	0.43	0.74	1.4
% pasture per municipality with VHR imagery coverage	47	9.8	7	1.4	5.4	12	35

Supplementary Table S1: Summary statistics of the municipal-level analysis comparing scaled Congested Scene Recognition (CSR)-based estimation on very high resolution (VHR) images, and official cattle stocking rates for 47 municipalities in the Brazilian Amazon. 9 of the 56 municipalities were excluded from the municipal stocking rate analysis due to insufficient imagery coverage (<50 km^2 pasture). Official cattle head data from the Brazilian Institute of Geography and Statistics (IBGE) annual agricultural survey (Pesquisa Pecuária Municipal, PPM; 2018–2019) were combined with total pasture area from MapBiomas for each municipality to compute official stocking rates (cattle head/ pasture area in hectares). For the CSR-based analysis, pasture areas were derived by cropping MapBiomas pasture maps to the corresponding satellite imagery outlines. The CSR-derived stocking rates were scaled by a factor of 1.3. Both animals located inside and outside property boundaries were counted. Imagery coverage is key to a robust comparison: The years 2018 and 2019 were combined to have a higher imagery coverage per municipality. Municipalities with less than 50 km^2 of pasture detected in the available satellite imagery were excluded.

Supplementary Figure S3.

a

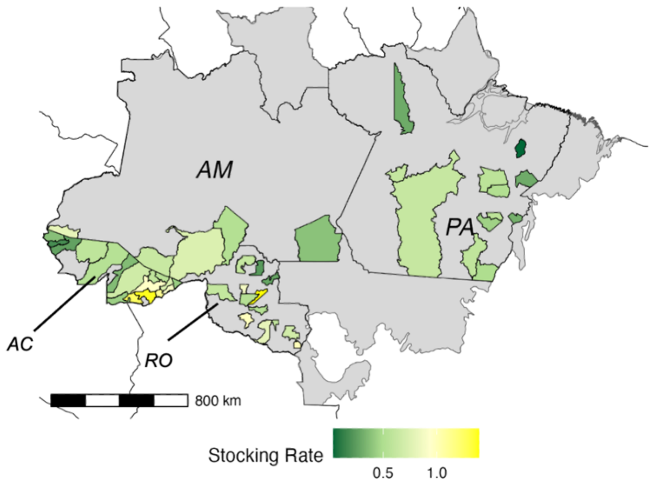

b

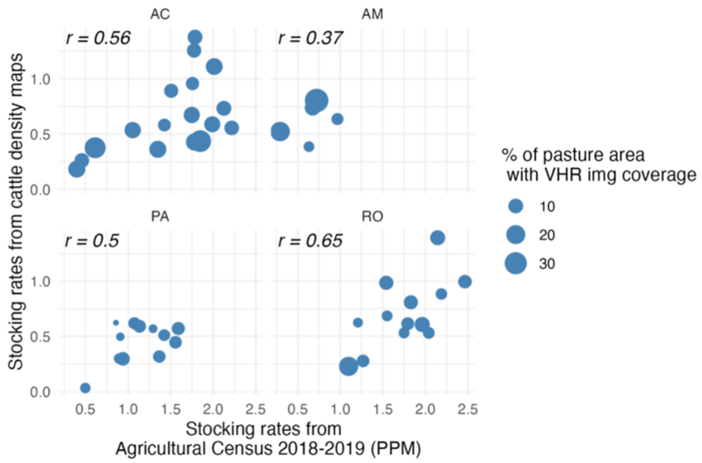

Supplementary Figure S3: Cattle density map derived municipal stocking rate estimates and agreement with official municipal stocking rate estimates. (a) Municipal stocking rates derived from cattle density maps (b-d) Pearson correlation r between municipality-level stocking rates (average of 2018-2019 official census data from Pesquisa Pecuária Municipal, PPM) and cattle density maps. Only municipalities with more than 50 km² of pasture detected in the available satellite imagery were included, as smaller coverage was insufficient for robust municipal-level estimates, resulting in a total of 150 satellite image outlines and 47 municipalities. The years 2018 and 2019 were combined to have a higher coverage per municipality. To calculate pasture areas, pasture maps from MapBiomas have been cropped to satellite imagery outlines. The correlation coefficient (r) is displayed for each state: for Acre (AC) r = 0.56, for Amazonas (AM) r = 0.37, for Pará (PA) r = 0.5 and for Rondônia (RO) r = 0.65, indicating a moderate positive relationship. Dot size represents the percentage of the municipality covered by available satellite imagery (larger dots indicate more comprehensive satellite data for that municipality, potentially leading to more reliable estimates). The overall Pearson correlation is 0.56.

Supplementary Figure S4.

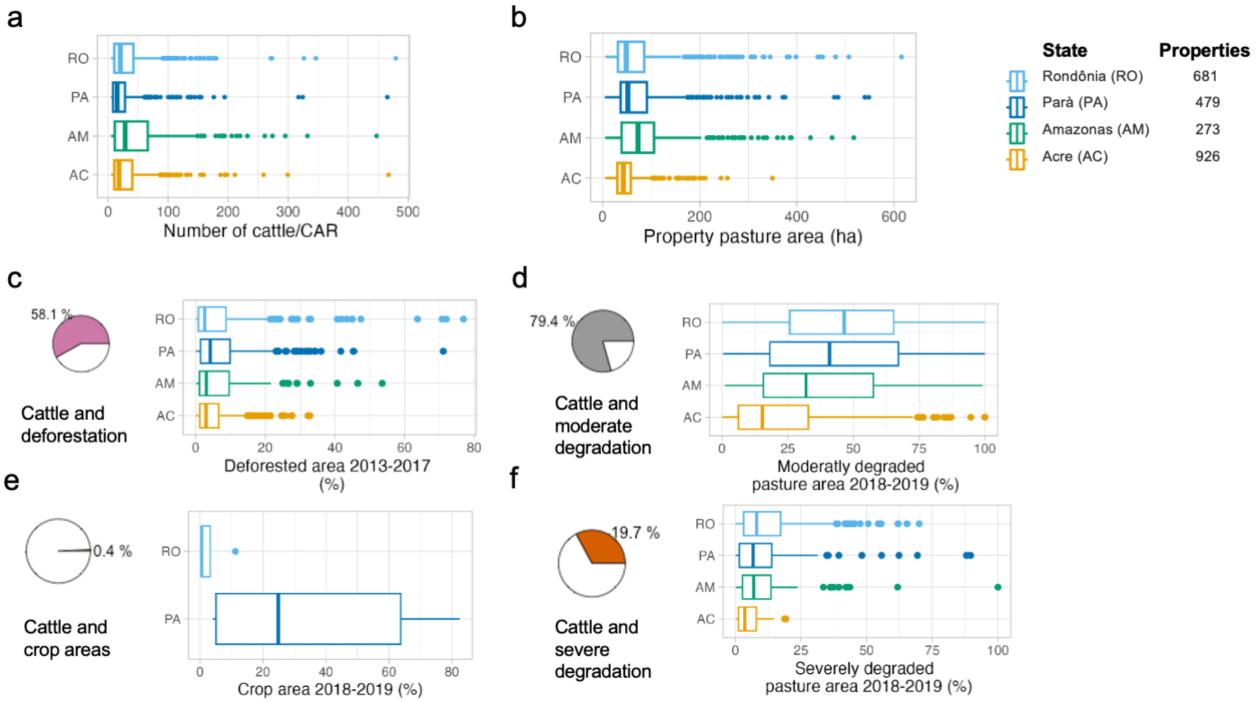

Supplementary Figure S4: Land management characteristics of properties with cattle recorded on satellite images in 2018-2019 in Acre (AC), Amazonas (AM), Pará (PA), and Rondônia (RO). (a) Scaled number of cattle on the properties (CAR= Cadastro Ambiental Rural, Rural Environmental Registry form from which property boundaries were retrieved) and (b) pasture areas of the properties are analyzed alongside land management variables: (c) Deforestation, (d) moderate pasture degradation, (e) crop plantations, and (f) severe pasture degradation. For each land management variable (c-f), pie charts illustrate the percentage of properties with observed cattle where more than 0.01 ha of that variable was recorded. Boxplots display the distribution of each of these land management variables (including the number of cattle in (a) and pasture area in (b)) across the properties. Each boxplot shows the median (center line), upper and lower quartiles (box boundaries), whiskers extending up to 1.5 times the interquartile range, and individual points indicating outliers.

Supplementary Table S2.

Variable	Description	Data source	*URL*	Unit	Period
Stocking rate	Number of cattle from ensemble CSR predictor / pasture	-	-	Animal/pasture area (ha)	2018-2019 for FE-Full model and Figure S4
Pasture	Area of pasture (class ID 15)	MapBiomas v7	https://brasil.mapbiomas.org/en/map/colecao-7/	ha	Mean of 2018-2019
Deforestation for pasture	Area of forest changing to pasture (class ID 315).	MapBiomas v7	"	% of Property Area	2013-2017
Forest Cover	Area of forest (class ID 3)	MapBiomas v7	"	% of Property Area	2013
Forest in 10 km buffer	Area of forest (class ID 3) in a 10 km buffer zone around property	MapBiomas v7	"	% of total buffer area	2013
Crop area	Area covered by agricultural area (class ID 39,41)	MapBiomas v7	"	ha	Mean of 2018-2019
Pasture degradation	Area of moderate (class ID 2) and severe pasture degradation (class ID 1)	MapBiomas v7	"	ha	Mean of 2018-2019
Distance to nearest federal slaughterhouse	Distance from property to the nearest slaughterhouse	Trase	https://trase.earth	km	2021
Population Density	Population density	IBGE	IBGE via Sidrar API for R.	Inhabitants/ Municipality Area	2018-2019
Precipitation	Mean monthly precipitation	NASA's Monthly Global Precipitation Measurement	https://developers.google.com/earth-engine/datasets/catalog/NASA_GPM_L3_IMERG_MONTHLY_V06	mm/hr (municipality level)	Mean of 2018-2019
Temperature	Mean monthly temperature	NASA's Global Change Observation Mission	https://developers.google.com/earth-engine/datasets/catalog/JAXA_GCOM-C_L3_LAND_LST_V2	°C (municipality level)	Mean of 2018-2019
Historic Stocking Rates	Stocking Rates derived from the Municipal Survey (Pesquisa Pecuária Municipal)	IBGE/ MapBiomas v7	IBGE via Sidrar API for R.	Animals/pasture Area (municipality level)	2013
Property boundaries and property area	Rural Environmental Registry - Cadastro Ambiental Rural (CAR)	SICAR	https://www.car.gov.br/	ha	Accessed in August 2022

Supplementary Table S2: Data description for variables used in summary statistics and regression model, including a description, data source, unit, and period.

Supplementary Table S3.

Variable	*N*	*Mean*	*Std. Dev.*	*Min*	*Pctl. 25*	*Pctl. 75*	*Max*
State	2359						
... Pará	479	20%					
... Rondônia	681	29%					
... Acre	926	39%					
... Amazonas	273	12%					
Number of cattle	2359	33	42	5	9	40	479
Uncertainty	2359	0.11	0.066	0	0.063	0.16	0.29
Pasture area [ha]	2359	66	64	4.5	33	74	616
Stocking rate	2359	0.73	0.63	0.02	0.31	0.94	6.7
Deforestation 2013-2017 [%]	2359	4.1	7.2	0	0	4.8	74
Distance to nearest federal sl aughterhouse [10km]	2359	8.5	6.8	0.59	4.1	11	46
Property Size [ha]	2359	92	91	6	48	99	766
Pesquisa Pecuaria Municipal st ocking rate (2013, [municipality -level])	2359	1.5	0.5	0.26	1.1	1.8	2.5
Forest in 10 km buffer (2013, %)	2359	40	19	25	26	54	93
Forest cover (2013, %)	2359	28	24	0	6.7	44	100
Avg. precipitation (2018–2019, mm/hr)	2359	0.37	0.072	0.19	0.33	0.43	0.5
Avg. temperature (2018–2019, °C)	2359	33	1.5	30	31	34	37
Population density (2018–2019, n/km²)	2359	6.8	6.1	0.6	2.9	10	68
Deforestation (2013–2014, %)	2359	1.1	3.1	0	0	0.83	60
Deforestation (2014–2015, %)	2359	1.4	3.6	0	0	1.1	62
Deforestation (2015–2016, %)	2359	1.1	2.8	0	0	0.79	32
Deforestation (2016–2017, %)	2359	0.45	1.3	0	0	0.17	16

Supplementary Table S3: **Summary Statistics for Properties in Acre, Amazonas, Rondônia, and Pará used in the Regression Analysis, and Additional Variables including 2359 properties**. The variables used within the Fixed Effects-Full regression model are shown in bold. The table also includes other relevant variables for a broader context of the property characteristics.

Supplementary Table S4.

	Main (FE)	*No FE*	*FE + Heteroskedasticity-robust SE*	*Timing controls*	*FE + Timing controls + Hetero SE*
Deforestation (2013–2017, %)	-0.009**	-0.010**	-0.009***		
	(0.003)	(0.003)	(0.002)		
Distance to nearest slaughterhouse (10 km)	-0.013**	-0.011**	-0.013***	-0.013**	-0.013***
	(0.004)	(0.004)	(0.002)	(0.004)	(0.002)
Property size (ha)	-0.001***	-0.001***	-0.001***	-0.001***	-0.001***
	(0.000)	(0.000)	(0.000)	(0.000)	(0.000)
PPM stocking rate (2013, municipality-level)	-0.088	0.074	-0.088*	-0.087	-0.087*
	(0.096)	(0.063)	(0.038)	(0.096)	(0.038)
Forest in 10 km buffer (2013, %)	-0.157	-0.200	-0.157+	-0.161	-0.161+
	(0.135)	(0.166)	(0.095)	(0.137)	(0.095)
Forest cover (2013, %)	0.002*	0.003*	0.002**	0.003*	0.003**
	(0.001)	(0.001)	(0.001)	(0.001)	(0.001)
Avg. precipitation (2018–2019)	-1.015*	0.772*	-1.015***	-1.003*	-1.003***
	(0.481)	(0.342)	(0.269)	(0.482)	(0.270)
Avg. temperature (2018–2019)	-0.081*	-0.062*	-0.081***	-0.080*	-0.080***
	(0.032)	(0.031)	(0.016)	(0.032)	(0.016)
Population density (2018–2019)	9.049	7.181	9.049**	9.108	9.108**
	(5.824)	(6.392)	(3.305)	(5.845)	(3.311)
Deforestation (2013–2014, %)				-0.005	-0.005
				(0.004)	(0.004)
Deforestation (2014–2015, %)				-0.007*	-0.007*
				(0.003)	(0.003)
Deforestation (2015–2016, %)				-0.011*	-0.011**
				(0.004)	(0.004)
Deforestation (2016–2017, %)				-0.024***	-0.024**
				(0.006)	(0.008)
Num.Obs.	2359	2359	2359	2359	2359
R2	0.094	0.074	0.094	0.095	0.095
RMSE	0.60	0.61	0.60	0.60	0.60
Std.Errors	by: municipality	by: municipality	Heteroskedasticity-robust	by: municipality	Heteroskedasticity-robust

Supplementary Table S4: Regression results for the relationship between deforestation, forest cover, and cattle stocking density under alternative model specifications. Column (1) reports the main specification with fixed effects and standard errors clustered at the municipality level; Column (2) omits fixed effects; Column (3) includes fixed effects with heteroskedasticity-robust standard errors; Column (4) adds timing controls by disaggregating deforestation into annual periods (2013–2017). Column (5) combines fixed effects, timing controls, and heteroskedasticity-robust standard errors. Reported coefficients are followed by standard errors in parentheses. Significance levels: + $p < 0.1$, * $p < 0.05$, ** $p < 0.01$, *** $p < 0.001$.

Supplementary Table S5.

	Main (FE)	*No FE*	*FE + Heteroskedasticity-robust SE*	*Timing controls*	*FE + Timing controls + Hetero SE*
Deforestation (2013–2017, %)	-0.007**	-0.007**	-0.007***		
	(0.002)	(0.002)	(0.001)		
Distance to nearest slaughterhouse (10 km)	-0.010**	-0.008**	-0.010***	-0.010**	-0.010***
	(0.003)	(0.003)	(0.002)	(0.003)	(0.002)
Property size (ha)	-0.000***	-0.001***	-0.000***	-0.000***	-0.000***
	(0.000)	(0.000)	(0.000)	(0.000)	(0.000)
PPM stocking rate (2013, municipality-level)	-0.068	0.057	-0.068*	-0.067	-0.067*
	(0.074)	(0.049)	(0.029)	(0.074)	(0.029)
Forest in 10 km buffer (2013, %)	-0.121	-0.154	-0.121+	-0.124	-0.124+
	(0.104)	(0.127)	(0.073)	(0.106)	(0.073)
Forest cover (2013, %)	0.002*	0.002*	0.002**	0.002*	0.002**
	(0.001)	(0.001)	(0.001)	(0.001)	(0.001)
Avg. precipitation (2018–2019)	-0.780*	0.594*	-0.780***	-0.771*	-0.771***
	(0.370)	(0.263)	(0.207)	(0.371)	(0.208)
Avg. temperature (2018–2019)	-0.062*	-0.048*	-0.062***	-0.062*	-0.062***
	(0.025)	(0.023)	(0.012)	(0.025)	(0.012)
Population density (2018–2019)	0.007	0.006	0.007**	0.007	0.007**
	(0.004)	(0.005)	(0.003)	(0.004)	(0.003)
Deforestation (2013–2014, %)				-0.004	-0.004
				(0.003)	(0.003)
Deforestation (2014–2015, %)				-0.005*	-0.005*
				(0.002)	(0.002)
Deforestation (2015–2016, %)				-0.008*	-0.008**
				(0.003)	(0.003)
Deforestation (2016–2017, %)				-0.018***	-0.018**
				(0.005)	(0.006)
Num.Obs.	2359	2359	2359	2359	2359
R2	0.094	0.074	0.094	0.095	0.095
RMSE	0.46	0.47	0.46	0.46	0.46
Std.Errors	by: municipality	by: municipality	Heteroskedasticity-robust	by: municipality	Heteroskedasticity-robust

Supplementary Table S5: Regression results for unscaled stocking density and relationship between deforestation, forest cover, and cattle under alternative model specifications. Column (1) reports the main specification with fixed effects and standard errors clustered at the municipality level; Column (2) omits fixed effects; Column (3) includes fixed effects with heteroskedasticity-robust standard errors; Column (4) adds timing controls by disaggregating deforestation into annual periods (2013–2017). Column (5) combines fixed effects, timing controls, and heteroskedasticity-robust standard errors. Reported coefficients are followed by standard errors in parentheses. Significance levels: + $p < 0.1$, * $p < 0.05$, ** $p < 0.01$, *** $p < 0.001$. The coefficients are smaller, however, as expected, the significance of the estimates remains stable compared to the scaled stocking density in Table S7.
