# Supplementary material for: Spatial patterns of cattle densities across the Brazilian Amazon revealed by very high-resolution satellite imagery
Source: Commun Sustain. 2026 Jun 16;1(1):98. doi: 10.1038/s44458-026-00082-2 (PMC13271885; doi:10.1038/s44458-026-00082-2)
Supplement: Supplementary file 3 — Description of Additional Supplementary Files [file 44458_2026_82_MOESM3_ESM.pdf]

Description of Additional Supplementary Files

File name: Supplementary data S1

Description: Cattle density estimates derived from very high-resolution (VHR) satellite imagery. The georeferenced data includes Congested Scene Recognition-based predictions of cattle counts (n_cattle) and their associated uncertainty (n_cattle_sd), as well as the VHR image date (img_date), the image patch identifier (id_bbox) at the image-tile level, across four states of the Brazilian Amazon.

File name: Supplementary data S2

Description: 170 VHR satellite images from 56 municipalities in the states of Acre, Amazonas, Pará, and Ron image geometries and assigned dates are also visible in the Google Earth Project.
