# Supplementary material for: Spatial patterns of cattle densities across the Brazilian Amazon revealed by very high-resolution satellite imagery
Source: Commun Sustain. 2026 Jun 16;1(1):98. doi: 10.1038/s44458-026-00082-2 (PMC13271885; doi:10.1038/s44458-026-00082-2)
Supplement: Supplementary file 1 — Peer review file [file 44458_2026_82_MOESM1_ESM.pdf]

nature portfolio

Peer Review File

Spatial patterns of cattle densities across the Brazilian Amazon revealed by very high-resolution satellite imagery

Corresponding Author: Dr Leonie Hodel

This file contains all editorial decision letters in order by version, followed by all author rebuttals in order by version.

This file contains all reviewer reports in order by version, followed by all author rebuttals in order by version.

This manuscript has been previously reviewed at another Nature Portfolio journal. This document only contains reviewer comments and rebuttal letters for versions considered at Communications Sustainability.

Version 0:

Decision Letter:

** Please ensure you delete the link to your author home page in this e-mail if you wish to forward it to your coauthors **

Dear Dr Hodel,

Your manuscript titled "Satellite imagery mapping reveals cattle density patterns and linked deforestation in the Brazilian Amazon" has now been seen by 1 reviewer, and we include their comments at the end of this message. In light of their advice we are pleased to say that we are interested in publishing a suitably revised version in Communications Sustainability, under the condition that you can make the minimum data set needed to replicate your results available to our readers, and clearly explain in the Data Availability Statement how the data can be obtained (on a case-by-case basis if not in a publicly accessible repository). We may have to obtain further reviewer advice on this point.

We therefore invite you to revise and resubmit your manuscript, along with a point-by-point response that takes into account the points raised. Please highlight all changes in the manuscript text file.

Please submit your point-by-point responses as a separate file, distinct from your cover letter where you can add responses to the Editors' comments that you do not want to be made available to the reviewers. Word files are preferred. We recommend that any figures, tables or graphs that are included in the response to reviewers are also included in the main article or Supplementary Information.

We are committed to providing a fair and constructive peer-review process. Please don't hesitate to contact us if you wish to discuss the revision in more detail.

Please use the following link to submit your revised manuscript, point-by-point response to the reviewers' comments (which should be in a separate document to any cover letter), a tracked-changes version of the manuscript (as a PDF file) and the completed checklist:
Link Redacted
** This url links to your confidential home page and associated information about manuscripts you may have submitted or be reviewing for us. If you wish to forward this email to co-authors, please delete the link to your homepage first **

We hope to receive your revised paper within six weeks; please let us know if you aren't able to submit it within this time so that we can discuss how best to proceed. If we don't hear from you, and the revision process takes significantly longer, we may close your file. In this event, we will still be happy to reconsider your paper at a later date, as long as nothing similar has been accepted for publication at Communications Sustainability or published elsewhere in the meantime.

Please do not hesitate to contact us if you have any questions or would like to discuss these revisions further. We look forward to seeing the revised manuscript and thank you for the opportunity to review your work.

Best regards,

Mengjie Wang

Consulting Editor, Communications Sustainability
Associate Editor, Communications Earth & Environment

EDITORIAL POLICIES AND FORMATTING

We ask that you ensure your manuscript complies with our editorial policies. Please ensure that the following formatting requirements are met, and any checklist relevant to your research is completed and uploaded as a Related Manuscript file type with the revised article.

For Manuscripts that fall into the following fields:
• Behavioural and social science
• Ecological, evolutionary & environmental sciences
• Life sciences
An updated and completed version of our Reporting Summary must be uploaded with the revised manuscript
You can download the form here:
https://www.nature.com/documents/nr-reporting-summary.pdf

Furthermore, please align your manuscript with our format requirements, which are summarized on the following checklist:
Communications Sustainability formatting checklist

and also in our style and formatting guide Communications Sustainability formatting guide .

*** DATA: Communications Sustainability endorses the principles of the Enabling FAIR data project (http://www.copdess.org/enabling-fair-data-project/). We ask authors to make the data that support their conclusions available in permanent, publicly accessible data repositories. (Please contact the editor if you are unable to make your data available).

All Communications Sustainability manuscripts must include a section titled "Data Availability" at the end of the Methods section or main text (if no Methods). More information on this policy, is available at http://www.nature.com/authors/policies/data/data-availability-statements-data-citations.pdf.

In particular, the Data availability statement should include:
- Unique identifiers (such as DOIs and hyperlinks for datasets in public repositories)
- Accession codes where appropriate
- If applicable, a statement regarding data available with restrictions
- If a dataset has a Digital Object Identifier (DOI) as its unique identifier, we strongly encourage including this in the Reference list and citing the dataset in the Data Availability Statement.

DATA SOURCES: All new data associated with the paper should be placed in a persistent repository where they can be freely and enduringly accessed. We recommend submitting the data to discipline-specific, community-recognized repositories, where possible and a list of recommended repositories is provided at http://www.nature.com/sdata/policies/repositories.

If a community resource is unavailable, data can be submitted to generalist repositories such as figshare or Dryad Digital Repository. Please provide a unique identifier for the data (for example a DOI or a permanent URL) in the data availability statement, if possible. If the repository does not provide identifiers, we encourage authors to supply the search terms that will return the data. For data that have been obtained from publicly available sources, please provide a URL and the specific data product name in the data availability statement. Data with a DOI should be further cited in the methods reference section.

Please refer to our data policies at http://www.nature.com/authors/policies/availability.html.

REVIEWER COMMENTS:

Reviewer #2 (Remarks to the Author):

The authors have improved the technical descriptions in the paper and acknowledged their limitations in the revised version. Other minor issues are listed below.

- P.23, l.118-119: “map” by definition is (for example) “a graphic representation, drawn to scale and usually on a flat surface, of features” (https://www.britannica.com/science/map). Please simply don’t call them maps if you are not producing maps.

- Figure 2b: I meant Fig2b has some darker blue colored polygons and light-grey polygons on the map. But the legend depicts a blue color for "within property boundaries", which does not even exist on the map. Not nitpicking --- a map should be consistent in its symbolic representation and its legend. Once again, the authors should carefully CHECK everything!!! I've spotted too many mistakes since the first round of review.

- P.10, l.424: the previous version stated "four counted animals", and now in this new version, the threshold suddenly changed to "a minimum of five animals"???

- In the response letter, the authors stated "Existing evidence from our datasets and prior work indicates that more than 90% of forest loss in the study region is followed by conversion to pasture". Any reference? Any methodological assumptions or arguments like this should be clearly stated in the main text for better scientific reporting.

** Visit Nature Portfolio's author and reviewers' website at www.nature.com/authors for information about policies, services and author benefits**

Communications Sustainability is committed to improving transparency in authorship. As part of our efforts in this direction, we are now requesting that all authors identified as 'corresponding author' create and link their Open Researcher and Contributor Identifier (ORCID) with their account on the Manuscript Tracking System prior to acceptance. ORCID helps the scientific community achieve unambiguous attribution of all scholarly contributions. You can create and link your ORCID from the home page of the Manuscript Tracking System by clicking on 'Modify my Springer Nature account' and following the instructions in the link below. Please also inform all co-authors that they can add their ORCIDs to their accounts and that they must do so prior to acceptance.
https://www.springernature.com/gp/researchers/orcid/orcid-for-nature-research

For more information please visit http://www.springernature.com/orcid

If you experience problems in linking your ORCID, please contact the Platform Support Helpdesk.

Version 1:

Decision Letter:

** Please ensure you delete the link to your author home page in this e-mail if you wish to forward it to your coauthors **

Dear Dr Hodel,

Your manuscript titled "Satellite imagery mapping reveals cattle density patterns and linked deforestation in the Brazilian Amazon" has now been seen by our reviewers, whose comments appear below. In light of their advice we are delighted to say that we are happy, in principle, to publish a suitably revised version in Communications Sustainability.

We therefore invite you to revise your paper one last time to address the remaining concerns of our reviewers. At the same time we ask that you edit your manuscript to comply with our format requirements and to maximise the accessibility and therefore the impact of your work.

EDITORIAL REQUESTS:

Please review our specific editorial comments and requests regarding your manuscript in the attached "Editorial Requests Table".

*****Please take care to match our formatting and policy requirements. We will check revised manuscript and return manuscripts that do not comply. Such requests will lead to delays. *****

Please outline your response to each request in the right hand column. Please upload the completed table with your manuscript files as a Related Manuscript file.

If you have any questions or concerns about any of our requests, please do not hesitate to contact me.

SUBMISSION INFORMATION:

In order to accept your paper, we require the files listed at the end of the Editorial Requests Table; the list of required files is also available at https://www.nature.com/documents/commsj-file-checklist.pdf .

OPEN ACCESS:

Communications Sustainability is a fully open access journal. Articles are made freely accessible on publication. For further information about article processing charges, open access funding, and advice and support from Nature Portfolio, please visit https://www.nature.com/commssustain/open-access

At acceptance, you will be provided with instructions for completing the open access licence agreement on behalf of all authors. This grants us the necessary permissions to publish your paper. Additionally, you will be asked to declare that all required third party permissions have been obtained, and to provide billing information in order to pay the article-processing charge (APC).

Please use the following link to submit the above items:
Link Redacted
** This url links to your confidential home page and associated information about manuscripts you may have submitted or be reviewing for us. If you wish to forward this email to co-authors, please delete the link to your homepage first **

We hope to hear from you within two weeks; please let us know if you need more time.

Best regards,

Mengjie Wang

Consulting Editor, Communications Sustainability
Associate Editor, Communications Earth & Environment

REVIEWERS' COMMENTS:

** Visit Nature Portfolio's author and reviewers' website at www.nature.com/authors for information about policies, services and author benefits**

Version 2:

Decision Letter:

Dear Dr Hodel,

We are delighted to accept your manuscript titled "Spatial patterns of cattle densities across the Brazilian Amazon revealed by very high-resolution satellite imagery" for publication in Communications Sustainability. Thank you for choosing to publish your interesting work with us.

Acceptance of your manuscript is conditional on all authors' agreement with our publication policies. In particular, your manuscript must not be published elsewhere and there must be no announcement of the work in the media until the publication date.

We welcome the submission of material for the ‘Featured Image’ section of the Communications Sustainability home page. Images should relate to the content of your manuscript, but do not need to be taken directly from the accepted work. Suggestions should be sent by email to commssustain@nature.com, along with the completed Licence to Publish form. Please note that images should be supplied as1400x400-pixel, in RGB. Unfortunately, we cannot promise that your suggestions will be used.

Please carefully read the information below for information on what to expect from the next steps of the publishing process:

If you are planning an embargoed press release or require a specific publication date, please complete our scheduling requests form, or contact commsproduction@springernature.com, as soon as possible after acceptance and we will endeavour to accommodate your request.

For further information on the journey of your article from acceptance to publication, please see our Author FAQs.

Publishing Agreements and Fees:

In about one week, you will receive an email with a link to complete the appropriate grant of rights necessary for publishing your paper and – if applicable – to provide payment information for your article-processing charge (APC), either via credit card or by requesting an invoice.

If needed, our Author Services team will be in touch regarding any additional information that may be required.

In order to avoid any delays, please ensure that you have emails from Springer Nature whitelisted in your mail system.

If you have any questions about our publishing options, costs, Open Access requirements, or our legal forms, please contact ASJournals@springernature.com

Proofs:

At this point we will also edit your manuscript to ensure that it conforms with our house style. Once you have completed the publication agreement and arranged payment, you will receive a separate email with a link to an online eProof for you to review. Please read your proof with great care to ensure that no changes have been introduced which have inadvertently altered the meaning of your paper. We suggest that you discuss the proof with your co-authors, but please ensure that only one author communicates with us and that only one set of corrections is returned via the online correction in the eProof.

The corresponding (or nominated) author is responsible on behalf of all co-authors for the accuracy of all content, including spelling of names and current affiliations.

To ensure prompt publication, your proofs should be returned within two working days. If there is any period within the next four weeks in which you won't be available, please nominate a co-author with whom we can correspond and send their contact information to us via email at commsproduction@springernature.com as soon as possible.

Please note that your Supplementary Information files are now finalised, and they will be submitted as provided for preparation for publication of the Article. Any requests to make changes will only be considered in exceptional circumstances and will result in a delay to publication.

You will not receive access to your eProof until the Licence to Publish and Article-Processing Charge steps are completed.

Providing great service is very important to us. We would greatly appreciate any comments you have about your experience at Communications Sustainability. We look forward to publishing your paper, and we hope to work with you again in the future.

Best regards,
Mengjie Wang

Consulting Editor, Communications Sustainability
Associate Editor, Communications Earth & Environment

*You can now use a single sign-on for all your accounts, view the status of all your manuscript submissions and reviews, access usage statistics for your published articles and download a record of your reviewing activity for the Nature Portfolio journals. Please check your account regularly and ensure that we have your current contact information.

** Visit Nature Research's author and referees' website at www.nature.com/authors for information about policies, services and author benefits**

We may promote your article on social media once it is published, so please feel free to send me the twitter handles of any authors or departments and we will be sure to tag them accordingly.

Open Access This Peer Review File is licensed under a Creative Commons Attribution 4.0 International License, which permits use, sharing, adaptation, distribution and reproduction in any medium or format, as long as you give appropriate credit to the original author(s) and the source, provide a link to the Creative Commons license, and indicate if changes were made.
In cases where reviewers are anonymous, credit should be given to 'Anonymous Referee' and the source.
The images or other third party material in this Peer Review File are included in the article's Creative Commons license, unless indicated otherwise in a credit line to the material. If material is not included in the article's Creative Commons license and your intended use is not permitted by statutory regulation or exceeds the permitted use, you will need to obtain permission directly from the copyright holder.
To view a copy of this license, visit https://creativecommons.org/licenses/by/4.0/

Reviewer #2 (Remarks to the Author):

The authors have improved the technical descriptions in the paper and acknowledged their limitations in the revised version.

Thank you very much for revising the manuscript and providing these additional comments. We are very grateful for your time and efforts to improve the manuscript.

Other minor issues are listed below.

- P.23, l.118-119: “map” by definition is (for example) “a graphic representation, drawn to scale and usually on a flat surface, of features” (https://www.britannica.com/science/map). Please simply don’t call them maps if you are not producing maps.

Great point. We did change that term from ‘cattle density maps’ to ‘cattle densities’ (l.118). We think that, since density is mass per unit area, calling it cattle density and noting a 120 m resolution is a more accurate description than ‘cattle density maps’.

- Figure 2b: I meant Fig2b has some darker blue colored polygons and light-grey polygons on the map. But the legend depicts a blue color for "within property boundaries", which does not even exist on the map. Not nitpicking --- a map should be consistent in its symbolic representation and its legend. Once again, the authors should carefully CHECK everything!!! I’ve spotted too many mistakes since the first round of review.

We do see the confusion here and acknowledge this point. Our aim is to depict both the property boundaries and the land cover simultaneously, which is why we decided to have the blue color to appear transparent. We have now changed the color in the legend accordingly, as well as the overlapping property boundaries.

- P.10, l.424: the previous version stated “four counted animals”, and now in this new version, the threshold suddenly changed to “a minimum of five animals”???

Thanks for highlighting this. This was a correction of a technical description to match the code implementation: We noticed that the hyperparameter was defined as the minimum number of animals being >4, rather than >=4, in the code. We accounted for that in the revised version of the manuscript.

- In the response letter, the authors stated “Existing evidence from our datasets and prior work indicates that more than 90% of forest loss in the study region is followed by conversion to pasture”. Any reference? Any methodological assumptions or arguments like this should be clearly stated in the main text for better scientific reporting.

We do reference this statement in the main text already (l. 62):

Our study region is the Brazilian Amazon, where more than 90% of deforested areas are converted into pasture (2,27).

This statement is supported by Haddad (2024) and by the reference to MapBiomas (2023), the underlying dataset used by Haddad and by us. Citing Haddad, 2024:

‘Cattle ranching was the main proximate cause, accounting for 93.4% of the regional total, followed by crops (mainly soybean, maize and cotton; 6.4%) and mining (0.2%).’ (p.1142).
